# The correlation between clinical outcomes and genomic analysis with high risk factors for the progression of osteosarcoma

**DOI:** 10.1002/1878-0261.13526

**Published:** 2023-10-04

**Authors:** Weifeng Liu, Huanqing Cheng, Zhen Huang, Yaping Li, Yanrui Zhang, Yongkun Yang, Tao Jin, Yang Sun, Zhiping Deng, Qing Zhang, Feng Lou, Shanbo Cao, Huina Wang, Xiaohui Niu

**Affiliations:** ^1^ Department of Orthopaedic Oncology Surgery, Beijing Jishuitan Hospital Peking University Beijing China; ^2^ Fourth Medical College of Peking University Beijing China; ^3^ National Center for Orthopeadics Beijing China; ^4^ Acornmed Biotechnology Co., Ltd. Beijing China

**Keywords:** actionable mutation profile, disease progression, molecular characterization, osteosarcoma, risk factors

## Abstract

Osteosarcoma (OS) is a rare but aggressive malignancy. Despite previous reports, molecular characterization of this disease is not well understood, and little is known regarding OS in Chinese patients. Herein, we analyzed the genomic signatures of 73 Chinese OS cases. *TP53*, *NCOR1*, *LRP1B*, *ATRX*, *RB1*, and *TFE3* were the most frequently mutated gene in our OS cohort. In addition, the genomic analysis of Western OS patients was performed. Notably, there were remarkable disparities in mutational landscape, base substitution pattern, and tumor mutational burden between the Chinese and Western OS cohorts. Specific molecular mechanisms, including DNA damage repair (DDR) gene mutations, copy number variation (CNV) presence, aneuploidy, and intratumoral heterogeneity, were associated with disease progression. Additionally, 30.1% of OS patients carried clinically actionable alterations, which were mainly enriched in PI3K, MAPK, DDR, and RTK signaling pathways. A specific molecular subtype incorporating DDR alterations and CNVs was significantly correlated with distant metastasis‐free survival and event‐free survival, and this correlation was observed in all subgroups with different characteristics. These findings comprehensively elucidated the genomic profile and revealed novel prognostic factors in OS, which would contribute to understanding this disease and promoting precision medicine of this population.

## Graphical abstract

Here, a distinctive genomic profile was revealed in Chinese patients with osteosarcoma (OS). Specific molecular mechanisms were found to be associated with disease progression. Additionally, a novel molecular subtype incorporating DNA damage repair gene mutations and copy number variations was identified, which can effectively define the risk stratification of localized OS and better predict metastasis or recurrence.

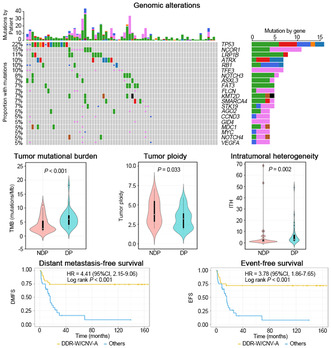

**Abbreviations**
AUC: area under the ROC curve
CCF: cancer cell fraction
CNV: copy number variation
DDR: DNA damage repair
DDR‐M/CNV‐A: DDR mutations/CNV absence
DDR‐M/CNV‐P: DDR mutations/CNV presence
DDR‐W/CNV‐A: DDR gene wild‐type/CNV absence
DDR‐W/CNV‐P: DDR gene wild‐type/CNV presence
DMFS: distant metastasis‐free survival
EFS: event‐free survival
ExAC: Exome Aggregation Consortium
INDEL: small insertion or deletion
ITH: intratumoral heterogeneity
OS: osteosarcoma
ROC: receiver operating characteristic
SNV: single‐nucleotide variant
TMB: tumor mutational burden
wGII: weighted‐genomic integrity index

## 1 Introduction

Osteosarcoma (OS) is the most frequent type of primary malignant bone tumor in children, adolescents, and young adults [1]. It most commonly occurs in the metaphysis of long bones such as the femur, tibia, and humerus [2]. The combination of systemic chemotherapy and surgery led to a 5‐year survival rate of ~ 70% in patients with localized disease. However, the 5‐year survival rate is only ~20% in patients with metastasis or relapse [3]. For localized OS, although the survival has reached a plateau since the introduction of systemic cytotoxic chemotherapy, around 30–40% of patients still experience metastasis or recurrence within 2–3 years after completing treatment [3]. Increasing data have revealed the association between genomic features and clinical outcomes in multiple malignancies, whereas limited information is available on the association exploration in OS. This emphasizes the urgent need for molecular characterization and precision medicine research for OS.

In recent years, genome‐wide profiling has greatly improved our knowledge of the molecular profiles of various tumors and facilitated the identification of new prognostic indicators or therapeutic targets [4, 5]. However, compared with other malignancies, only a few studies have investigated the genomic landscape of OS due to its low incidence. For instance, several previous studies revealed that the frequently altered driver genes in OS included *TP53*, *RB1*, *ATRX*, *RECQL4*, *PTEN*, *BRCA2*, insulin‐like growth factor (IGF) pathway genes, and other PI3K pathway members [6, 7, 8, 9]. Unfortunately, to date, genomic studies of OS have mainly been conducted in patients from Western countries. Although there have been limited studies reporting the genomic profile in Chinese OS, most of them had some limitations, including a low sample size, a small number of cancer‐related genes to be estimated, as well as a lack of investigation of prognostic molecular markers [10]. Therefore, systematic molecular profiling of Chinese OS is urgently needed. Therapeutic strategies have been established for OS over the past 20 years [11], and novel approaches for treatments and prevention need to be further developed. Unfortunately, few actionable therapeutic targets have been identified, and no further improvements in clinical outcomes have been made within the past decades due to the lack of available knowledge of the genomic profile of OS. Although surgery combined with chemotherapy has markedly improved the clinical outcome of patients with localized OS, many patients experience frequent local recurrence and/or distant metastasis after surgery, leading to an extremely poor prognosis. To our knowledge, the underlying molecular mechanisms related to disease progression in OS have not been elucidated. Therefore, it is of great importance to further systematically clarify the molecular features of OS to enable accurate stratification and treatment.

Herein, molecular profiling in a cohort of Chinese patients with OS was comprehensively performed. The present study elucidated the specific mutational signatures and actionable profile in OS patients, and further identified a molecular subtype that could redefine risk stratification and better predict metastasis and recurrence. Overall, these findings provide a comprehensive molecular basis and novel insights for understanding OS, which may help to differentiate OS patients with different prognosis and could play an important role in individualized treatment of this population in the future.

## 2 Materials and methods

### 2.1 Patients and sample collection

A total of 73 Chinese patients with OS from Beijing Jishuitan Hospital between September 2009 and September 2021 were enrolled in this study. Tumor and matched control blood samples were collected from these patients. The clinical stages ranged from II to III and were verified based on Enneking's surgical staging system. Genomic and clinical data for the Western OS cohort were acquired from cBioPortal [12]. These data were obtained from a previous study, which performed a comprehensive genomic analysis of 2138 sarcomas (including 129 OS patients) using Memorial Sloan Kettering‐Integrated Mutation Profiling of Actionable Cancer Targets (MSK‐IMPACT) assay [13]. After excluding OS patients whose race categories were unknown or who came from Far East Asian countries, a total of 93 Western patients with OS harboring genomic data were included in subsequent analyses. This study was approved by the ethical committee of the Beijing Jishuitan Hospital (JST‐K2023‐013‐00). Written informed consent was obtained from all participants. The study methodologies conformed to the standards set by the Declaration of Helsinki.

### 2.2 Library preparation and next‐generation sequencing

Tissue DNA and blood DNA were extracted utilizing the QIAamp Genomic DNA Kit (Qiagen, Hilden, Germany) based on the manufacturer's instructions. Sequencing libraries were constructed based on the protocols recommended by the Illumina TruSeq DNA Library Preparation Kit (Illumina Inc., San Diego, CA, USA). Briefly, genomic DNA was sheared into 150–250 bp fragments by Covaris M220 ultrasonoscope (Covaris, Woburn, MA, USA), and then fragmented DNA was end‐repaired, A‐tailed, and ligated to the adapters. Libraries were hybridized with the designed 808 gene panel, a custom SeqCap EZ Choice Library (Roche NimbleGen, Madison, WI, USA), which included genes (hotspot regions, selected exons, or complete coding regions) related to solid tumors (Table S1). The captured libraries were then pooled and sequenced on the HiSeq2500 NGS platform (Illumina Inc.). Average coverage depth for tumor tissues was 3284X. After removing the low‐quality sequencing data, the reads were aligned to the human reference genome (GRCh37) using the Burrows–Wheeler alignment tool [14]. Base recalibration was carried out by GATK v3.8. Single‐nucleotide variants (SNVs) and small insertions or deletions (INDELs) were identified by MuTect2 v1.1.7 with the recommended parameters.

### 2.3 Tumor mutation burden analysis

Tumor mutational burden (TMB) is calculated as the number of somatic alterations per megabase (Mb) of genome examined, including synonymous SNVs, nonsynonymous SNVs, and INDELs. Variants with a mutant‐allele frequency of ≥ 0.5% in the Exome Aggregation Consortium (ExAC) database were excluded.

### 2.4 Estimation of weighted‐genomic integrity index, tumor ploidy, and intratumoral heterogeneity

gistic2.0 software, The broad institute of MIT and Harvard, Cambridge, MA, USA was applied to identify common copy number variant (CNV) regions, including chromosome arm‐level CNV regions and focal CNV regions [15]. To detect genes with CNV, the parameters were as follows: genegistic 1, smallmem 1, broad 1, brlen 0.5, and conf 0.90. All other parameters were set to default values. Weighted‐genomic integrity index (wGII), tumor ploidy, and intratumoral heterogeneity (ITH) analyses were performed based on absolute software (version 1.0.6) [16]. The wGII was calculated by the total length of gain regions plus loss regions divided by the chromosome size. Tumor ploidy analysis was performed by the absolute algorithm according to the CNV results [16]. The clonal or subclonal status of mutations was analyzed based on the value of the cancer cell fraction (CCF), which was the estimated fraction of tumor cells carrying this mutation in a sample. Mutations were categorized as clonal mutations if the evaluated CCF was more than 0.9 and the Pr (clonal) (representing the probability that a mutation is clonal) was > 0.5 and as subclonal mutations otherwise [17]. The ITH was defined as the ratio of the sum of subclonal mutation numbers to the sum of clonal mutation numbers.

### 2.5 Statistical analysis

Distant metastasis‐free survival (DMFS) was defined as the time from the pathologic diagnosis of OS to the first distant metastasis or the last follow‐up. Event‐free survival (EFS) was measured as the time from the pathologic diagnosis of OS to the first disease progression (locoregional recurrence and/or distant metastasis) or the last follow‐up. graphpad prism software (version 8.0.2), La Jolla, CA, USA and r software (version 4.1.3), Vienna, Austria were used to conduct the statistical analyses. Fisher's exact test or the chi‐squared test was carried out to evaluate the genomic differences between the Chinese and Western OS cohorts as well as between patients with and without disease progression. Comparisons of continuous variables between the two groups were determined by the Mann–Whitney *U*‐test. Univariate analysis was performed with the Kaplan–Meier method and the log‐rank test, and Multivariate analysis was conducted based on a Cox proportional hazards regression model. A two‐sided *P* < 0.05 was considered statistically significant.

## 3 Results

### 3.1 Patient characteristics

The clinical features of all the OS patients are summarized in Table S2. There were 23 females (31.5%) and 50 males (68.5%) with a median age of 19 years (range, 5–64 years). The vast majority of patients had clinical stage II disease (87.7%). Primary tumor lesions were in femur (36/73, 49.3%), tibia (20/73, 27.4%), humerus (8/73, 10.9%), ilium (2/73, 2.7%), thigh (2/73, 2.7%), fibula (1/73, 1.4%), calcaneus (1/73, 1.4%), rib (1/73, 1.4%), tarsus (1/73, 1.4%), and foot (1/73, 1.4%).

### 3.2 Genomic profile of OS

**Fig. 1:**
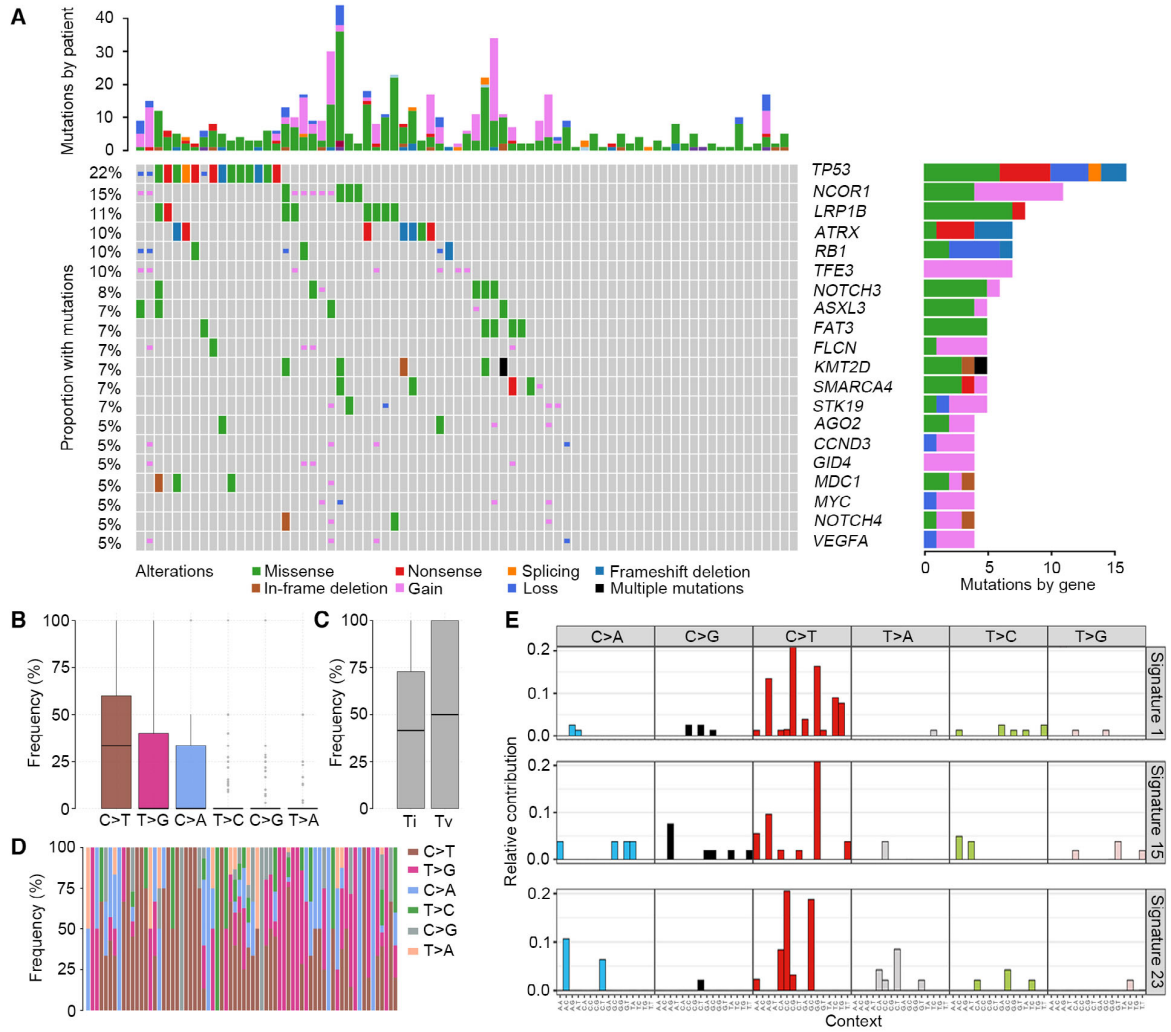
Molecular profile of Chinese patients with OS. (A) The mutational landscape of all cases. (B) The bar plot exhibits each type of transition (Ti) or transversion (Tv) in OS. Error bars represent standard deviation. (C) The bar plot shows overall Ti and Tv patterns in OS. Error bars represent standard deviation. (D) The box plot exhibits the distribution of each type of Ti or Tv for each patient. (E) Three independent mutational signatures identified in OS. OS, osteosarcoma.

Among all the patients, 98.6% (72/73) of them carried at least one genomic alteration, including SNVs, INDELs or CNVs, which was summarized in Table S3. The most frequently mutated genes in our series of OS were *TP53* (22%), *NCOR1* (15%), *LRP1B* (11%), *ATRX* (10%), *RB1* (10%), and *TFE3* (10%) (Fig. 1A). Among all the patients, 38.4% of them carried CNVs, and the most commonly amplified genes were *NCOR1* and *TFE3*, followed by *CCND3*, *FLCN*, and *GID4*. The most commonly deleted genes were *RB1*, *GSTT1*, and *TP53* (Fig. S1A). The most significant regions with amplifications were observed in 17p11.2 and 1q21.3 (Fig. S1B), and the most marked regions with deletions were found in 14q32.33 and 17p13.1 (Fig. S1C). TMB was defined as the number of somatic base substitutions or INDELs in coding regions per Mb. The median TMB was low (median: 4.9, range: 1.6–19.9 mutations·Mb^−1^). To further explore the molecular features of OS, base substitution and mutational signature analyses were performed. The C to T (C>T) transition (36.3%) was the predominant mutation type in Chinese OS, followed by T to G (T>G) and C to A (C>A) transversions (23.6% and 17.2%, respectively) (Fig. 1B). The profile of base substitutions showed that the frequency of transversion was higher than that of transition (Fig. 1C,D). Mutational signature analysis identified three independent mutational signatures that matched known COSMIC signatures 1, 15, and 23 (Fig. 1E). Signature 1 is known to be associated with age and is observed in multiple tumor types. Signature 15 is correlated with defective DNA mismatch repair and occurs in stomach cancers and small cell lung carcinoma. The etiology of signature 23 remains unknown, but it shows very strong transcriptional strand bias for C>T mutations. Given the finding that signature 15 was related to defective DNA mismatch repair, mutations in the DNA damage repair (DDR) signaling pathway were further investigated (Table S4). In our study, 23.3% of patients carried DDR mutations, and the most frequently altered genes were *MSH2*, *BLM*, *CDK12*, and *FANCE* (Fig. S2A). Numerous studies have revealed that patients harboring DDR mutations show high TMB in various cancer types, whereas the correlation between DDR mutations and TMB in OS has never been explored. Herein, patients with DDR alterations tended to show higher TMB than patients without DDR alterations (*P* = 0.055) (Fig. S2B).

Furthermore, a comparison of genomic landscape between juveniles (children/adolescents) and adults was performed. Notably, mutational profiles exhibited remarkable differences between the two groups. In the juvenile group, the most commonly mutated genes were *NCOR1*, *TP53*, and *ATRX* (Fig. S3A). In the adult group, the most frequently mutated genes were *TP53*, *RB1*, and *LRP1B* (Fig. S3B). Further statistical analysis revealed that the frequencies of *RB1* and *KMT2D* mutations in the adult group were significantly higher than those in the juvenile group (Fig. S3C). Additionally, TMB between juvenile and adult patients was compared. Unfortunately, there was no difference in the TMB between the two groups (Fig. S3D). CNV profile and DDR mutational profile between juvenile and adult tumors were found to be remarkably different (Fig. S4A–D). However, no difference was observed in the proportion of patients harboring DDR mutations or CNVs between the two groups (Fig. S4E,F).

### 3.3 Comparison of the mutational landscape between Chinese and Western OS

**Fig. 2:**
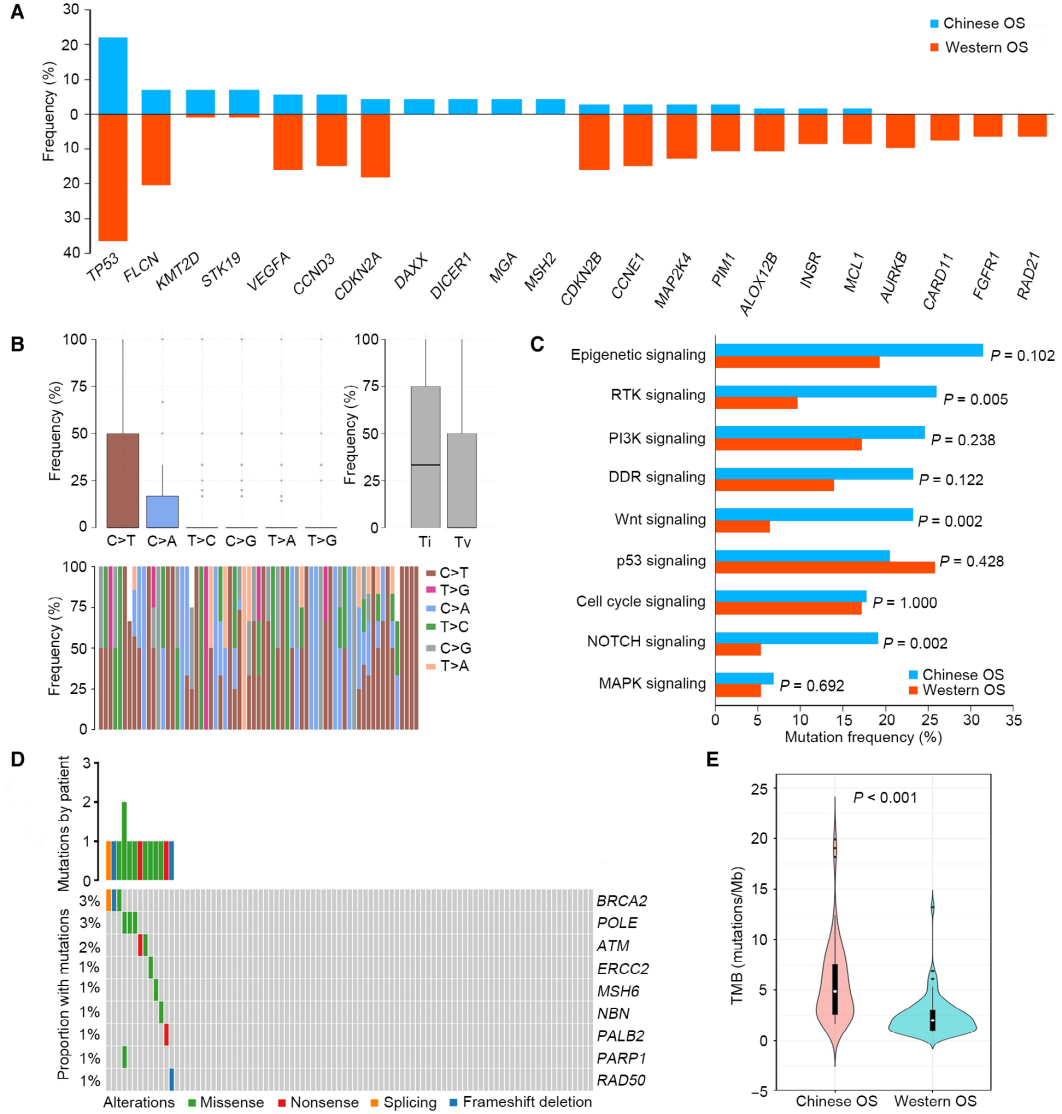
Comparison of genomic signatures between the Chinese and Western OS cohorts. (A) Significantly different mutated genes between the Chinese and Western OS cohorts. (B) The profile of base substitutions in Western OS. Error bars represent standard deviation. (C) Comparison of the rates of affected signaling pathways between the Chinese and Western OS cohorts. (D) The DDR mutational landscape in Western OS. (E) Comparison of TMB between the Chinese and Western OS cohorts. DDR, DNA damage repair; OS, osteosarcoma; TMB, tumor mutational burden.

To better understand the specific molecular characteristics of Chinese OS, a comprehensive comparison of mutational profiles between Chinese and Western OS patients was carried out. Compared with Western OS patients, significantly more genetic alterations in *KMT2D*, *STK19*, *DAXX*, *DICER1*, *MGA*, and *MSH2* were found, and significantly fewer mutations in *TP53*, *FLCN*, *VEGFA*, *CCND3*, *CDKN2A*, *CDKN2B*, *CCNE1*, *MAP2K4*, *PIM1*, *ALOX12B*, *INSR*, *MCL1*, *AURKB*, *CARD11*, *FGFR1*, and *RAD21* were identified among Chinese OS patients (*P* < 0.05) (Fig. 2A). Moreover, the profile of base substitutions between the two groups was further compared. Similar to Chinese OS, the C>T transition (31.7%) was still the dominant mutation type in Western OS. However, the frequency of the T>G transversion in Western OS was remarkably lower than that in Chinese OS (2.6% vs. 23.6%). In Western OS, the rate of transition was higher than that of transversion, which was opposite of that in Chinese OS (Fig. 2B). When exploring the differences in altered signaling pathways, we observed that the frequencies of the RTK, Wnt, and NOTCH signaling pathways between the two groups were significantly different (Fig. 2C). Notably, the proportion of affected DDR signaling pathway in Chinese OS was higher than that in Western OS (23.3% vs. 14.0%), but no significant difference was found (*P* = 0.122). The DDR mutational landscape between the two groups was further investigated. In Western OS, the most commonly mutated DDR genes were *BRCA2*, *POLE*, and *ATM*, which was remarkably different from that in Chinese OS (Fig. 2D and Fig. S2A). Furthermore, the TMB of Chinese OS was higher than that of Western OS (*P* < 0.001) (Fig. 2E).

### 3.4 Underlying mechanisms of disease progression

**Fig. 3:**
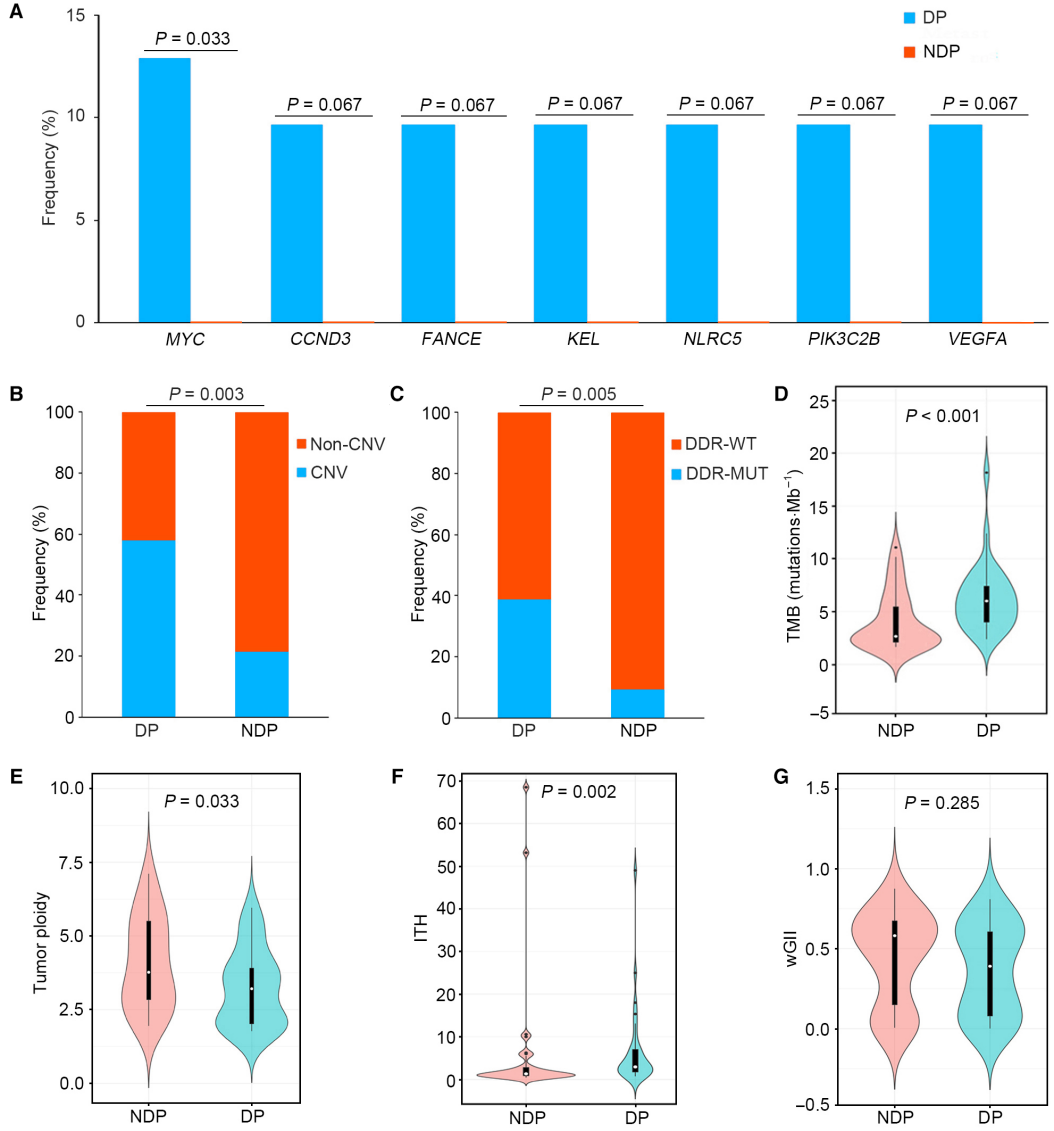
Underlying molecular mechanisms related to disease progression. Comparison of the frequencies of genomic mutations (A), rates of CNV presence (B), rates of DDR alterations (C), TMB (D), tumor ploidy (E), ITH (F), and wGII (G) between patients with and without disease progression. Significance was assessed by Chi‐square test (A–C) and Mann–Whitney test (D–G). CNV, copy number variation; DDR, DNA damage repair; DDR‐MUT, DNA damage repair gene mutations; DDR‐WT, DNA damage repair gene wild‐type; DP, patients with disease progression; ITH, intratumor heterogeneity; NDP, patients without disease progression; TMB, tumor mutational burden; wGII, weighted‐genomic integrity index.

In the whole group of patients, 64 (87.7%, 64/73) patients suffered from localized OS, who experienced disease progression in 31 cases during follow‐up. The genomic profiles between patients with and without disease progression were compared, and marked differences between them were found. In patients with disease progression, the most frequently mutated genes were *NCOR1* (23%), *TP53* (19%), *ATRX* (13%), *FAT3* (13%), *MYC* (13%), *NOTCH3* (13%), and *STK19* (13%) (Fig. S5A). However, in patients without disease progression, the most commonly mutated genes were *TP53* (27%) and *LRP1B* (12%) (Fig. S5B). Further statistical analysis showed that the frequency of *MYC* mutations in the group with disease progression was significantly higher than that in the group without disease progression (*P* = 0.033). *CCND3*, *FANCE*, *KEL*, *NLRC5*, *PIK3C2B*, and *VEGFA* alterations were more likely to occur in patients with disease progression (*P* = 0.067) (Fig. 3A). Additionally, the CNV profiles of the two groups were found to be different (Fig. S5C), and the proportion of patients harboring CNVs in the group with disease progression was strikingly higher than that in the group without disease progression (58.1% vs. 21.2%, *P* = 0.003) (Fig. 3B). Moreover, compared with patients without disease progression, DDR mutations were significantly enriched in patients with disease progression (*P* = 0.005) (Fig. 3C). Consistently, the TMB of the two groups exhibited a remarkable difference (*P* < 0.001) (Fig. 3D). Further analysis demonstrated that tumor ploidy was markedly lower in patients with disease progression than in patients without disease progression (*P* = 0.033) (Fig. 3E). In addition, there were significant differences in ITH between the two groups (*P* = 0.002) (Fig. 3F). Although the wGII in patients with disease progression was lower than that in patients without disease progression, no significant difference was observed (*P* = 0.285) (Fig. 3G).

### 3.5 Prognostic roles of genomic signatures

**Fig. 4:**
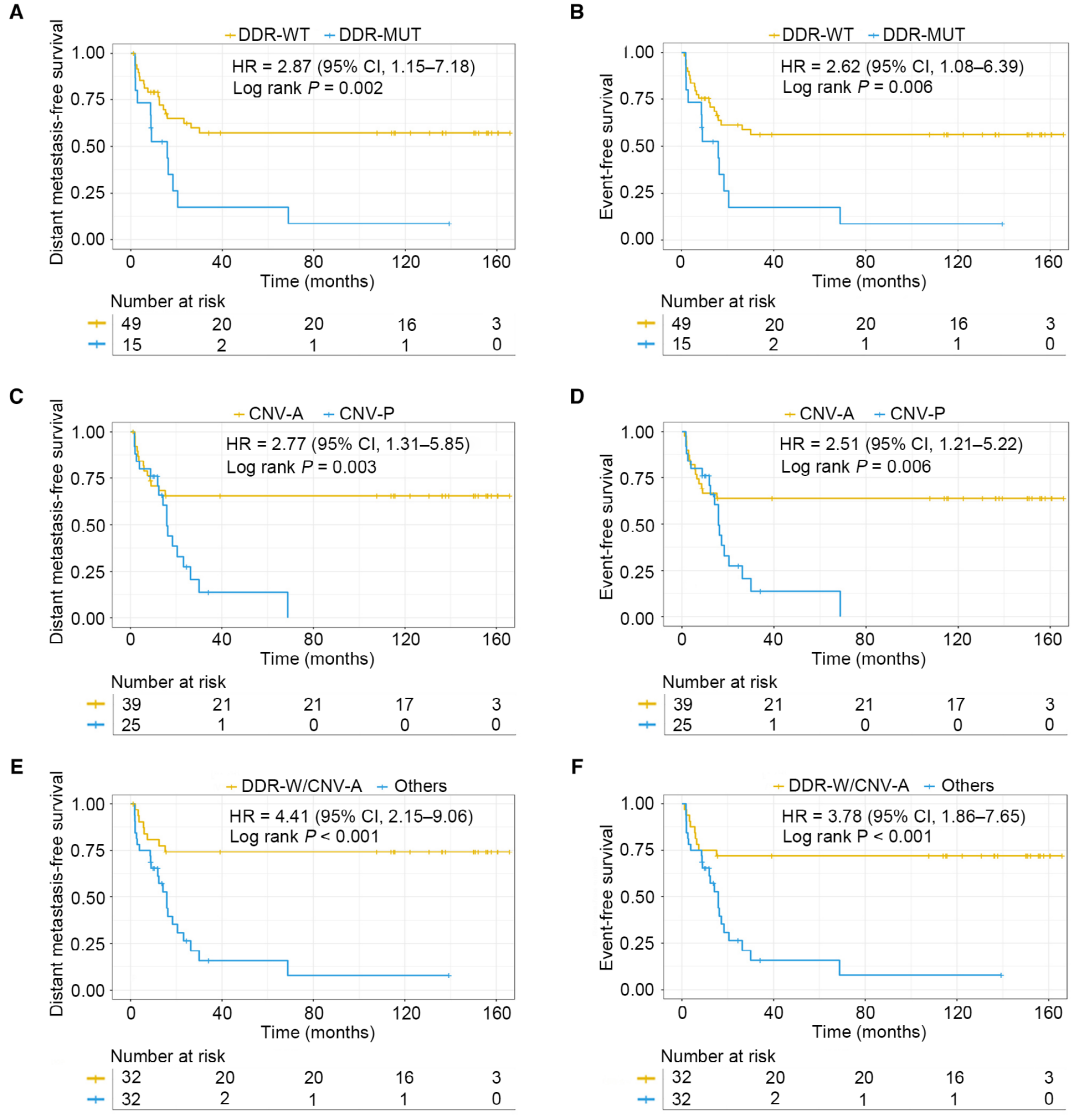
Effect of DDR mutations and CNV status on DMFS and EFS. (A, B) Kaplan–Meier estimates of DMFS and EFS for patients stratified by DDR mutation status. (C, D) Kaplan–Meier estimates of DMFS and EFS for patients stratified by CNV status. (E, F) Kaplan–Meier estimates of DMFS and EFS for patients stratified by the combination of DDR mutations and CNV status. CNV, copy number variation; CNV‐A, copy number variation absence; CNV‐P, copy number variation presence; DDR, DNA damage repair; DDR‐MUT, DNA damage repair gene mutations; DDR‐W/CNV‐A, DNA damage repair gene wild‐type/copy number variation absence; DDR‐WT, DNA damage repair gene wild‐type; DMFS, distant metastasis‐free survival; EFS, event‐free survival.

Due to the limited understanding of the association between molecular characteristics and OS biology, no studies have ever identified effective biomarkers related to recurrence and metastasis in patients with localized OS. For all the patients with localized OS in this study, DMFS and EFS data were collected, and the prognostic value of the molecular signatures was explored in depth. In our cohort, the median follow‐up time was 38.6 months, and 48.4% of patients experienced metastasis or recurrence during follow‐up. Patients with DDR mutations had worse DMFS and EFS than patients without DDR mutations (DDR WT) (hazard ratio (HR) = 2.87, 95% confidence interval (CI) = 1.15–7.18, *P* = 0.002 and HR = 2.62, 95% CI = 1.08–6.39, *P* = 0.006, respectively) (Fig. 4A,B). Distant metastasis occurred in 12 of 15 patients (80.0%) who harbored DDR mutations, while 19 of 49 patients (38.8%) with DDR WT experienced distant metastasis (Fig. S6A). The event rates between patients with and without DDR mutations were 80.0% and 40.8%, respectively (Fig. S6B). In addition, patients with CNVs also exhibited shorter DMFS and EFS compared with patients without CNVs (HR = 2.77, 95% CI = 1.31–5.85, *P* = 0.003 and HR = 2.51, 95% CI = 1.21–5.22, *P* = 0.006, respectively) (Fig. 4C,D). Distant metastasis rates in patients with and without CNVs were 72.0% and 33.3%, respectively (Fig. S6C). Patients with CNVs had a higher event rate than those without CNVs (72.0% vs. 35.9%) (Fig. S6D).

Next, DDR mutations and CNVs were combined into a single group, and their predictive prognostic value was explored. All patients were categorized into four subgroups: DDR mutations/CNV presence (DDR‐M/CNV‐P), DDR mutations/CNV absence (DDR‐M/CNV‐A), DDR WT/CNV presence (DDR‐W/CNV‐P), and DDR WT/CNV absence (DDR‐W/CNV‐A). Among the four groups, the DDR‐W/CNV‐A group showed the best DMFS and EFS (*P* < 0.001 and *P* = 0.002, respectively) (Fig. S7A,B). Apart from the DDR‐W/CNV‐A group, no marked significant differences in DMFS or EFS were found among the other three groups (*P* = 0.770 and *P* = 0.786, respectively) (Fig. S7C,D). Further analysis demonstrated that patients carrying DDR mutations and/or CNVs showed significantly worse DMFS and EFS than patients with DDR‐W/CNV‐A (HR = 4.41, 95% CI = 2.15–9.06, *P* < 0.001 and HR = 3.78, 95% CI = 1.86–7.65, *P* < 0.001, respectively) (Fig. 4E,F), which remarkably improved the metastasis and recurrence risk estimates compared with DDR mutations or CNVs alone. Distant metastasis occurred in 25.0% of patients with DDR‐W/CNV‐A, whereas 71.9% of patients with DDR mutations and/or CNVs had distant metastasis (Fig. S6E). Patients with DDR‐W/CNV‐A also had a lower event rate compared with the rest of the patients (28.1% vs. 71.9%) (Fig. S6F).

**Fig. 5:**
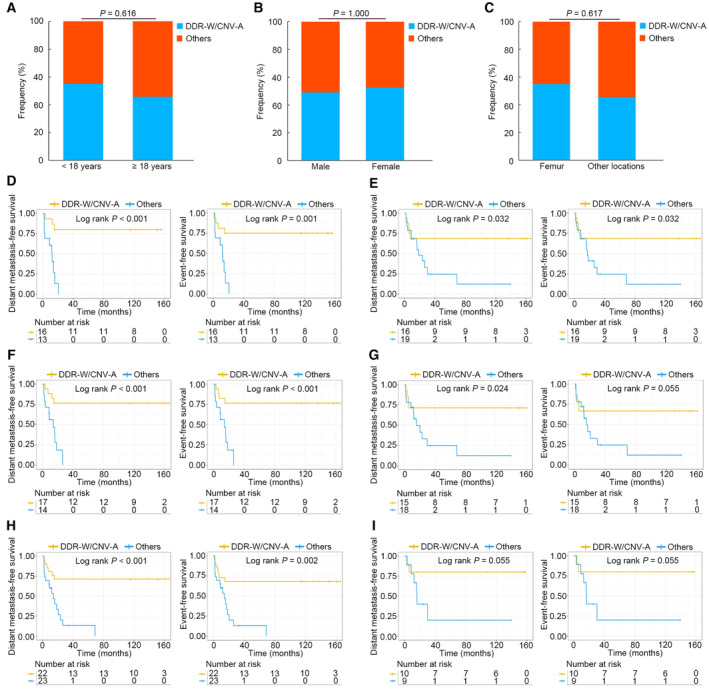
Correlation between the subtype incorporating DDR mutations and CNV status and the clinical characteristics, and the subgroup analysis of the clinical data. (A–C) The correlation between the subtype and age, sex, and primary tumor locations. Significance was assessed by Chi‐square test. (D) Kaplan–Meier estimates of DMFS and EFS for patients < 18 years of age. (E) Kaplan–Meier estimates of DMFS and EFS for patients ≥ 18 years of age. (F) Kaplan–Meier estimates of DMFS and EFS for patients with femur lesions. (G) Kaplan–Meier estimates of DMFS and EFS for patients with nonfemur lesions. (H) Kaplan–Meier estimates of DMFS and EFS for male patients. (I) Kaplan–Meier estimates of DMFS and EFS for female patients. CNV, copy number variation; DDR, DNA damage repair; DDR‐W/CNV‐A, DNA damage repair gene wild‐type/copy number variation absence; DMFS, distant metastasis‐free survival; EFS, event‐free survival.

To further verify the predictive ability for the prognosis of the molecular subtype, the correlation between this subtype and clinical characteristics was analyzed. The results showed that this subtype was not related to age, sex, and primary tumor locations (Fig. 5A–C). At the same time, a subgroup analysis of the clinical data of the different risk groups was performed in patients with different clinical features, which exhibited significant differences in the prognosis based on different age groups (< 18 years and ≥ 18 years) (Fig. 5D,E) and different tumor location groups (femur and other locations) (Fig. 5F,G). In different sex groups (male and female), a significant difference was observed in the prognosis of male patients (Fig. 5H), and a potentially statistically significant difference in the prognosis was found in female patients (*P* = 0.055) (Fig. 5I).

**Fig. 6:**
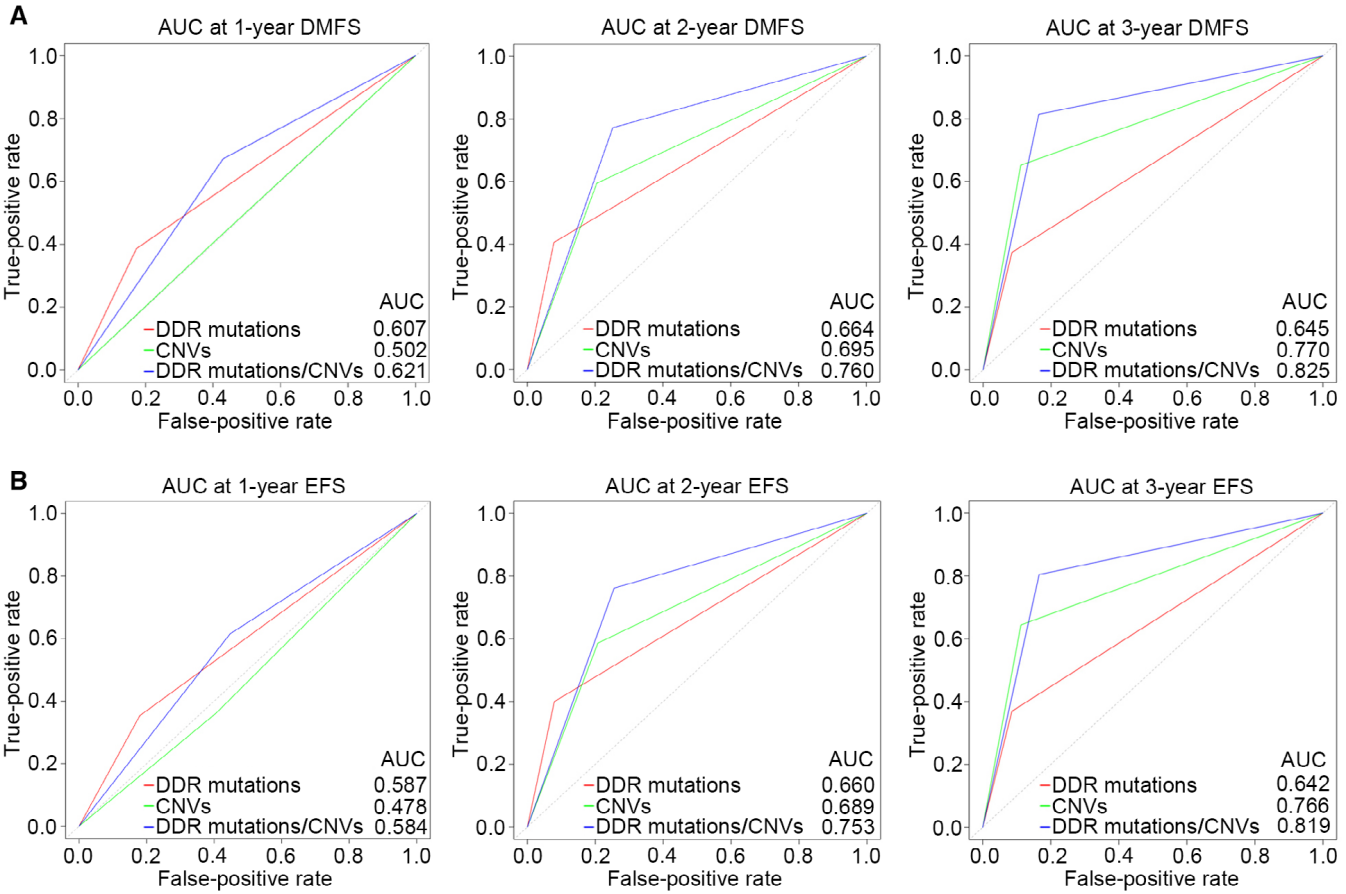
Time‐dependent ROC analysis of DMFS and EFS of different molecular subtypes. ROC curves for 1‐, 2‐, and 3‐year DMFS (A) and EFS (B) of DDR mutations, CNVs, and the newly defined risk stratification. AUC, area under the ROC curve; CNV, copy number variation; DDR, DNA damage repair; DMFS, distant metastasis‐free survival; EFS, event‐free survival; ROC, receiver operating characteristic.

In addition, we determined whether the molecular subtype was an independent risk factor for prognosis. After taking into account sex, age, and primary tumor location, the molecular subtype remained an independent prognostic factor for DMFS and EFS (HR = 5.41, 95% CI = 2.24–13.09, *P* < 0.001 and HR = 4.47, 95% CI = 1.93–10.37, *P* < 0.001, respectively) (Tables S5 and S6). Furthermore, time‐dependent receiver operating characteristic (ROC) curves were generated to compare the predictive abilities of DDR mutations, CNVs, and the redefined risk stratification according to the combination of the two predictive factors for DMFS and EFS. In the ROC analysis for 1‐, 2‐, and 3‐year DMFS prediction, redefined risk categorization showed a higher predictive significance than DDR mutations or CNVs alone (Fig. 6A). In the ROC analysis for 1‐year EFS prediction, the area under the curve (AUC) values of redefined risk categorization and DDR mutations were comparable. However, in the ROC analysis for 2‐ and 3‐year EFS prediction, redefined risk categorization had an AUC value of 0.753 and 0.819, respectively, which can still better predict recurrence or metastasis than DDR mutations or CNVs alone (Fig. 6B). Moreover, compared with other clinical characteristics, the specific molecular subtype of DDR mutations combined with CNVs had the highest AUC values of the ROC curves predicting DMFS and EFS (Fig. S8A,B), which further indicated its superior predictive ability.

### 3.6 Analysis of clinically actionable mutations

**Fig. 7:**
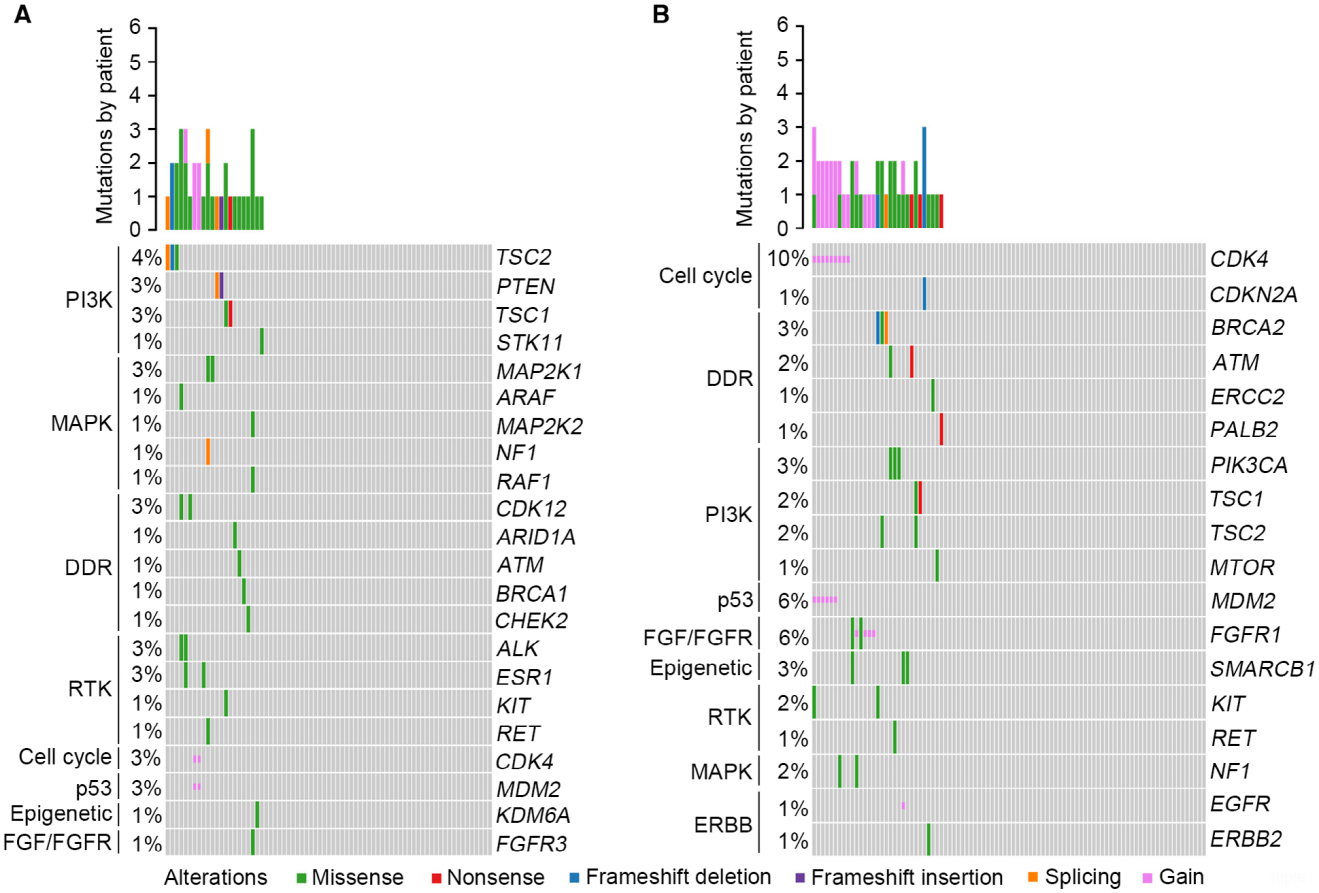
Overview of actionable alterations in OS. (A) Landscape of potentially targetable alterations in Chinese OS cohort. (B) Landscape of potentially targetable alterations in Western OS cohort. OS, osteosarcoma.

Based on the OncoKB classification system [18], the profile of clinically actionable alterations between Chinese and Western OS was analyzed and compared. In total, 34 potential drug‐related targets were identified from 22 Chinese OS patients (30.1%). Among patients with actionable mutations, 13 (59.1%) patients had one drug‐sensitive mutation, 6 (27.3%) had two, and 3 (13.6%) had three (Fig. S9A). The most frequent targets were mutations in *TSC2* (4%), *PTEN* (3%), *TSC1* (3%), *MAP2K1* (3%), *CDK12* (3%), *ALK* (3%), *ESR1* (3%), *CDK4* (3%), and *MDM2* (3%). Further analysis revealed that all actionable mutations were mainly enriched in the PI3K, MAPK, DDR, and RTK signaling pathways (Fig. 7A). Notably, the actionable profile of Western OS was remarkably different from that of Chinese OS. In Western OS patients, 49 clinically actionable mutations were detected in 31 patients (33.3%). However, the most commonly identified variants occurred in *CDK4* (10%), *MDM2* (6%), *FGFR1* (6%), *BRCA2* (3%), *PIK3CA* (3%), and *SMARCB1* (3%) for this group. All actionable alterations were primarily concentrated in the cell cycle, DDR, PI3K, p53, and FGF/FGFR signaling pathways (Fig. 7B). For Western OS patients carrying actionable alterations, 15 (48.4%) patients had one drug‐sensitive mutation, 14 (45.2%) had two, and 2 (6.5%) had three (Fig. S9B).

## 4 Discussion

Given the rarity of OS, the molecular characterization of this population is still unclear. In the present study, an in‐depth and comprehensive analysis of the genomic signatures of Chinese patients with OS was performed, and a specific molecular subtype associated with recurrence and metastasis was identified. Our study provides novel and important insights for understanding OS biology and promoting personalized medicine in this population.

Several previous studies conducted in OS patients from Western countries revealed that *TP53*, *RB1*, and *ATRX* were most commonly mutated [6, 7, 8]. Notably, in addition to these genes, we discovered frequent mutations in *NCOR1*, *LRP1B*, and *TFE3* in our cohort. *NCOR1* is a transcriptional coregulator that participates in bridging chromatin‐modifying enzymes with transcription factors and then affects the final transcriptional output [19]. Transcriptional coregulators are the major players in regulating epigenetic processes [20], implying a vital role of epigenetic signaling pathways in OS. *LRP1B* acts as a tumor suppressor gene encoding low‐density lipoprotein receptor [21] and is frequently altered in many types of cancer, including non‐small cell lung cancer, esophageal cancer, uterine cancer, and bladder cancer [22, 23]. However, to date, no research has reported a high prevalence of *LRP1B* mutations in OS. According to the analysis of the mutation data from a previously large Western OS cohort study [9], we found a high mutation frequency of *LRP1B* (6.3%), but it was not described in the study. Moreover, the rate of *LRP1B* mutation in our OS cohort was higher than that in the study OS cohort (11.0% vs. 6.3%), further indicating a high prevalence of *LRP1B* mutation in OS. Although the functional role of *LRP1B* dysfunction is still unclear, numerous studies have demonstrated that epigenetic mechanisms can affect *LRP1B* inactivation in several types of cancers [24, 25]. This further indicates the important role of epigenetic processes in OS carcinogenesis. *TFE3* is one of the members of the microphthalmia‐associated transcription factors (MITF) that act as master factors involved in regulating lysosomal biogenesis, autophagy, as well as immune responses [26]. Due to their functions among multiple cellular processes, dysregulation of these factors has been demonstrated to be correlated with many types of cancers or metabolic syndromes. Increasing studies have revealed that *TFE3* activation is required to maintain high levels of lysosomal function which is critical for tumor growth [27, 28], highlighting the potentially crucial function of *TFE3* in OS biology. Mutational signatures contribute to elucidating the complexity of mutational processes that occur during tumorigenesis. In this study, we found an enrichment of mutational signatures 1, 15, and 23 in our cohort. Signature 1 indicates an age‐related mutational process and has been found in all cancer types, which was consistent with other OS studies [9]. Signature 15 was associated with defective DNA mismatch repair and was commonly observed in stomach and small cell lung cancers, whereas it was never discovered in OS. Signature 23 shows very strong transcriptional strand bias for C>T substitutions, which was consistent with the profile of base substitutions observed in this study. To our knowledge, the current study is the first to report that signature 15 and signature 23 were the dominant mutational signatures in OS patients. The DDR system is required for genomic stability, and mutations in DDR genes generally lead to DDR deficiency [29]. The enrichment of signature 15 indicated the critical role of DDR deficiency in the development of OS.

To further explore the unique molecular features related to Chinese OS, a comparison of somatic substitutions between Chinese OS and Western OS was performed. Previous studies have discovered that C>T changes at CpG sites were the predominant mutation type in Western OS [6, 7]. This phenomenon still existed in Chinese OS, while the ratio of the T>G substitution in Chinese OS was strikingly higher than that in Western OS. The frequency of transition was lower than that of transversion in Chinese OS, but the opposite was observed in Western OS. Furthermore, the statistical analysis showed that a large number of genes had strikingly different mutation frequencies between the two groups. Regarding signaling pathways, we observed that the RTK, Wnt, and NOTCH pathways were more frequently affected in Chinese OS than in Western OS. Therefore, even though there was a similarity in the mutational profiles between Chinese and Western OS, a specific mutational pattern still existed in Chinese patients with OS. Herein, TMB was markedly higher in Chinese OS than in Western OS. Numerous reports have revealed that DDR mutations are associated with high TMB in various malignancies [30, 31], whereas the relationship between TMB and DDR mutations is unclear in OS. In the present study, we revealed that DDR alterations were potentially correlated with high TMB. It is noteworthy that the incidence of DDR mutations in Chinese OS was higher than that in Western OS, which might be one of the explanations for the TMB difference between the two groups. Increasing reports have demonstrated that patients with DDR alterations or high TMB could benefit from PD‐1/PD‐L1 blockade in many types of cancers [32, 33]. Despite the rare use of immune checkpoint inhibitor (ICI) treatments in OS, an increasing number of preclinical studies of immunotherapy have obtained promising results [34, 35]. Our findings implied that Chinese OS patients might be more likely to benefit from ICI treatments than Western OS patients. Currently, the targeted genomic profile for OS is rarely revealed due to the limited knowledge of the mutational landscape, especially in Chinese OS. The present study found that 30.1% of Chinese OS patients harbored at least one actionable mutation, suggesting the great significance of systematic molecular testing and the necessity of ‘basket’ clinical trials in which patients can participate based on their clinically actionable mutations. Among patients with actionable alterations, 40.9% of them carried at least two actionable mutations. This indicates that a combination of corresponding drugs might be useful to improve the outcome of this population in the future. The proportion of Western OS patients carrying clinically actionable mutations was similar to that of Chinese OS patients. However, the profile of clinically actionable alterations showed significant differences between the two groups. This suggests that deciphering the differences in the molecular characteristics of OS between different populations is conducive to understanding the mechanisms of carcinogenesis, improving precision therapy, and expanding the benefits of the population.

In the present study, 48.4% of patients with localized OS who underwent surgery experienced disease progression at the final follow‐up. To further understand the underlying mechanisms correlated with disease progression in OS, we systematically investigated the molecular differences between patients with disease progression and those without disease progression. According to the statistical analysis, several altered genes correlated with disease progression were identified, such as *MYC* and *VEGFA*. *MYC* is one of the most frequently altered oncogenes in human cancers, and its activation generally enhances cellular growth and survival [36, 37]. Numerous studies have revealed that *MYC* amplification is associated with metastasis and poor clinical outcomes in OS [38, 39]. Our results further confirmed the role of *MYC* activation during OS progression. Angiogenesis is crucial for the progression of multiple malignancies since the growth of tumors relies on vascular nutrition [40]. *VEGFA* is one of the angiogenic factors widely involved in the regulation of tumor angiogenesis and progression [41]. Yang et al. [42] revealed that *VEGFA* activation was significantly associated with adverse tumor‐free survival in OS patients, which further demonstrated the function of *VEGFA* in OS progression. Davoli et al. [43] revealed that aneuploidy, also known as somatic copy number alterations, is common in human malignancies and participates in driving tumorigenesis. Interestingly, in our study, patients with disease progression harbored higher levels of CNV and aneuploidy than patients without disease progression. Davoli et al. [43] found that patients with high CNV levels exhibited elevated expression of cell cycle and cell proliferation markers, and these patients generally had poor outcomes. These results imply that CNVs strikingly drive disease progression in OS. Genomic instability is a critical feature of cancer and plays a vital role in tumorigenesis as well as tumor progression [44]. Notably, the proportion of DDR alterations in patients with disease progression was significantly higher than that in patients without disease progression. This indicated that patients with disease progression might have a higher degree of genomic instability because of the deficiency of DNA strand break repair and chromosome maintenance, which was further verified by our finding that the wGII was relatively low in this population. Recently, Rong et al. [45] revealed that DDR alterations were strikingly associated with high mutant‐allele tumor heterogeneity in breast cancer. Additionally, this study showed that patients with disease progression presented with higher ITH than patients without disease progression. All these results further demonstrate the relationships between DDR gene mutations and high heterogeneity and rapid disease progression.

For localized OS, although ~ 70% of patients can be cured by current therapies incorporating surgical resection and combinational chemotherapy, a substantial number of patients still experience relapse or metastasis even with standard therapies [46]. Therefore, effective biomarkers are needed to better define the personalized risk of recurrence or metastasis and improve the clinical outcomes of patients with localized OS. Due to the low prevalence of OS and a lack of understanding its genomic profile, limited studies have simultaneously deciphered the molecular characterization and prognostic factors of OS. To date, no studies have explored biomarkers to define the recurrence or metastasis risk of patients with localized OS after surgery. Herein, we investigated in depth the molecular features related to recurrence and metastasis. The results showed that patients with DDR mutations had significantly poorer outcomes than those without DDR mutations. Further analysis showed that the presence of CNVs was also a prognostic predictor of poor outcomes. Overall, these results were consistent with the aforementioned findings that DDR alterations and CNVs were associated with disease progression in OS. Moreover, the prognostic value of DDR mutations combined with CNVs was further analyzed. We found that a specific molecular subtype of DDR‐W/CNV‐A was significantly correlated with improved DMFS and EFS. We also performed a subgroup analysis of the clinical data of different risk groups classified by the subtype, which revealed that the subtype had remarkable predictive ability for the prognosis of OS patients with different clinical features. Moreover, multivariate Cox regression analysis revealed that the molecular subtype was an independent predictive prognostic factor. Time‐dependent ROC analyses of DMFS and EFS further demonstrated that the redefined risk stratification based on the combination of two factors (DDR mutations and CNVs) is a much better prognostic factor than each alone. To the best of our knowledge, this study is the first to identify an effective molecular subtype that can define a subset of patients who experience a high risk of recurrence or metastasis after the completion of standard treatments in localized OS.

## 5 Conclusions

This study systematically depicted the molecular characterization of Chinese patients with OS and comprehensively performed a genomic landscape comparison between Chinese and Western OS, as well as between patients with disease progression and without progression, which laid the foundation for a deeper understanding of this rare cancer. Moreover, our findings provided supportive evidence for developing novel treatment strategies to improve the clinical outcomes of OS. For the first time, our study discovered that a specific molecular subtype of DDR mutations combined with CNVs can effectively define the risk stratification of localized OS. These findings will be of great importance in improving the individualized treatment and clinical management of OS in the future.

## Conflict of interest

The authors declare no conflict of interest.

## Author contributions

XN, WL, and HW conceived and designed the study. ZH, YY, TJ, YS, ZD, and QZ enrolled the patients, collected the corresponding clinical information, and followed up all participants. HC, YL, YZ, FL, and SC performed the sequencing and data analysis. WL wrote the paper. XN and HW participated in the revision of the draft. All authors contributed to the article and approved the final version.

### Peer review

The peer review history for this article is available at https://www.webofscience.com/api/gateway/wos/peer‐review/10.1002/1878‐0261.13526.

## Supporting information


**Fig. S1.** CNV landscape in Chinese patients with OS. (A) Mutation frequencies of CNVs among the top 20 genes. (B) Distribution of chromatin regions with amplifications. (C) Distribution of chromatin regions with deletions.
**Fig. S2.** DDR mutational profile and TMB analysis in Chinese OS. (A) DDR mutational profile in Chinese OS. (B) The comparison of TMB between patients with and without DDR mutations. DDR‐MUT, DNA damage repair gene mutations; DDR‐WT, DNA damage repair gene wild‐type.
**Fig. S3.** Comparison of the genomic landscape between juveniles (children/adolescents) and adults. (A) The mutational profile in juvenile patients. (B) The mutational profile in adult patients. (C) Comparison of the frequencies of genomic mutations between juvenile patients and adult patients. (D) Comparison of TMB between juvenile patients and adult patients.
**Fig. S4.** Comparison of DDR mutational profile and CNV profile between juveniles and adults. (A) The DDR mutational profile in juvenile patients. (B) The DDR mutational profile in adult patients. (C) The CNV profile in juvenile patients. (D) The CNV profile in adult patients. (E) Comparison of the rates of DDR alterations between juvenile patients and adult patients. (F) Comparison of the rates of CNV presence between juvenile patients and adult patients.
**Fig. S5.** Comparison of the genomic profiles between patients with and without disease progression. (A) The mutational landscape in patients with disease progression. (B) The mutational landscape in patients without disease progression. (C) The comparison of CNV landscape between patients with and without disease progression. DP, patients with disease progression; NDP, patients without disease progression.
**Fig. S6.** Correlation of DDR mutations and CNV status with distant metastasis rate and event rate. (A and B) Distant metastasis rate and event rate in patients stratified by DDR mutation status. (C and D) Distant metastasis rate and event rate in patients stratified by CNV status. (E and F) Distant metastasis rate and event rate in patients stratified by the combination of DDR mutations and CNV status. DDR‐WT, DNA damage repair gene wild‐type; DDR‐MUT, DNA damage repair gene mutations; CNV‐A, copy number variation absence; CNV‐P, copy number variation presence; DDR‐W/CNV‐A, DNA damage repair gene wild‐type/copy number variation absence; DM, distant metastasis; NDM, no distant metastasis; EO, event occurrence; NEO, no event occurrence.
**Fig. S7.** Effect of a combination of DDR mutations and CNVs on DMFS and EFS. (A and B) Kaplan–Meier estimates of DMFS and EFS for patients with four subgroups of DDR‐M/CNV‐P, DDR‐M/CNV‐A, DDR‐W/CNV‐P, and DDR‐W/CNV‐A. (C and D) Kaplan–Meier estimates of DMFS and EFS for patients with three subgroups of DDR‐M/CNV‐P, DDR‐M/CNV‐A, and DDR‐W/CNV‐P. DDR‐M/CNV‐P, DNA damage repair gene mutations/copy number variation presence; DDR‐M/CNV‐A, DNA damage repair gene mutations/ copy number variation absence; DDR‐W/CNV‐P, DNA damage repair gene wild‐type/copy number variation presence; DDR‐W/CNV‐A, DNA damage repair gene wild‐type/copy number variation absence.
**Fig. S8.** Time‐dependent ROC analysis of DMFS and EFS of different clinical and genomic features. ROC curves of 1‐, 2‐, and 3‐year DMFS (A) and EFS (B) among age, sex, tumor location, and the molecular subtype incorporating DDR mutations and CNVs.
**Fig. S9.** Frequency of cases with clinically actionable mutations in different OS cohorts. (A) Proportion of tumors carrying clinically actionable mutations in Chinese OS patients. (B) Proportion of tumors carrying clinically actionable mutations in Western OS patients.
**Table S1.** Gene lists and the corresponding targeted regions in the 808 cancer‐related gene panel.
**Table S2.** Clinical characteristics among 73 patients with osteosarcoma.
**Table S3.** All the genomic alterations identified in OS patients.
**Table S4.** Pathways and genes involved in the 808‐cancer‐gene panel.
**Table S5.** Univariate and multivariate analysis of factors associated with DMFS in OS patients.
**Table S6.** Univariate and multivariate analysis of factors associated with EFS in OS patients.

## Data Availability

All data in our study are available upon request.
