# Supplementary material for: The correlation between clinical outcomes and genomic analysis with high risk factors for the progression of osteosarcoma
Source: Mol Oncol. 2023 Oct 4;18(4):939–55. doi: 10.1002/1878-0261.13526 (PMC10994228; doi:10.1002/1878-0261.13526)
Supplement: Supplementary file 1 — Fig. S1. CNV landscape in Chinese patients with OS. (A) Mutation frequencies of CNVs among the top 20 genes. (B) Distribution of chromatin regions with amplifications. (C) Distribution of chromatin regions with deletions. Fig. S2. DDR mutational profile and TMB analysis in Chinese OS. (A) DDR mutational profile in Chinese OS. (B) The comparison of TMB between patients with and without DDR mutations. DDR‐MUT, DNA damage repair gene mutations; DDR‐WT, DNA damage repair gene wild‐type. Fig. S3. Comparison of the genomic landscape between juveniles (children/adolescents) and adults. (A) The mutational profile in juvenile patients. (B) The mutational profile in adult patients. (C) Comparison of the frequencies of genomic mutations between juvenile patients and adult patients. (D) Comparison of TMB between juvenile patients and adult patients. Fig. S4. Comparison of DDR mutational profile and CNV profile between juveniles and adults. (A) The DDR mutational profile in juvenile patients. (B) The DDR mutational profile in adult patients. (C) The CNV profile in juvenile patients. (D) The CNV profile in adult patients. (E) Comparison of the rates of DDR alterations between juvenile patients and adult patients. (F) Comparison of the rates of CNV presence between juvenile patients and adult patients. Fig. S5. Comparison of the genomic profiles between patients with and without disease progression. (A) The mutational landscape in patients with disease progression. (B) The mutational landscape in patients without disease progression. (C) The comparison of CNV landscape between patients with and without disease progression. DP, patients with disease progression; NDP, patients without disease progression. Fig. S6. Correlation of DDR mutations and CNV status with distant metastasis rate and event rate. (A and B) Distant metastasis rate and event rate in patients stratified by DDR mutation status. (C and D) Distant metastasis rate and event rate in patients stratified by CNV status. ( [file MOL2-18-939-s001.pdf]

The correlation between clinical outcomes and genomic analysis with high risk factors for the progression of osteosarcoma

Weifeng Liu[1,2,3], Huanqing Cheng[4], Zhen Huang[1,2,3], Yaping Li[4], Yanrui Zhang[4], Yongkun Yang[1,2,3], Tao Jin[1,2,3], Yang Sun[1,2,3], Zhiping Deng[1,2,3], Qing Zhang[1,2,3], Feng Lou[4], Shanbo Cao[4], Huina Wang[4,*] and Xiaohui Niu[1,2,3,*]

Supporting Information

Supplementary Figures and Tables

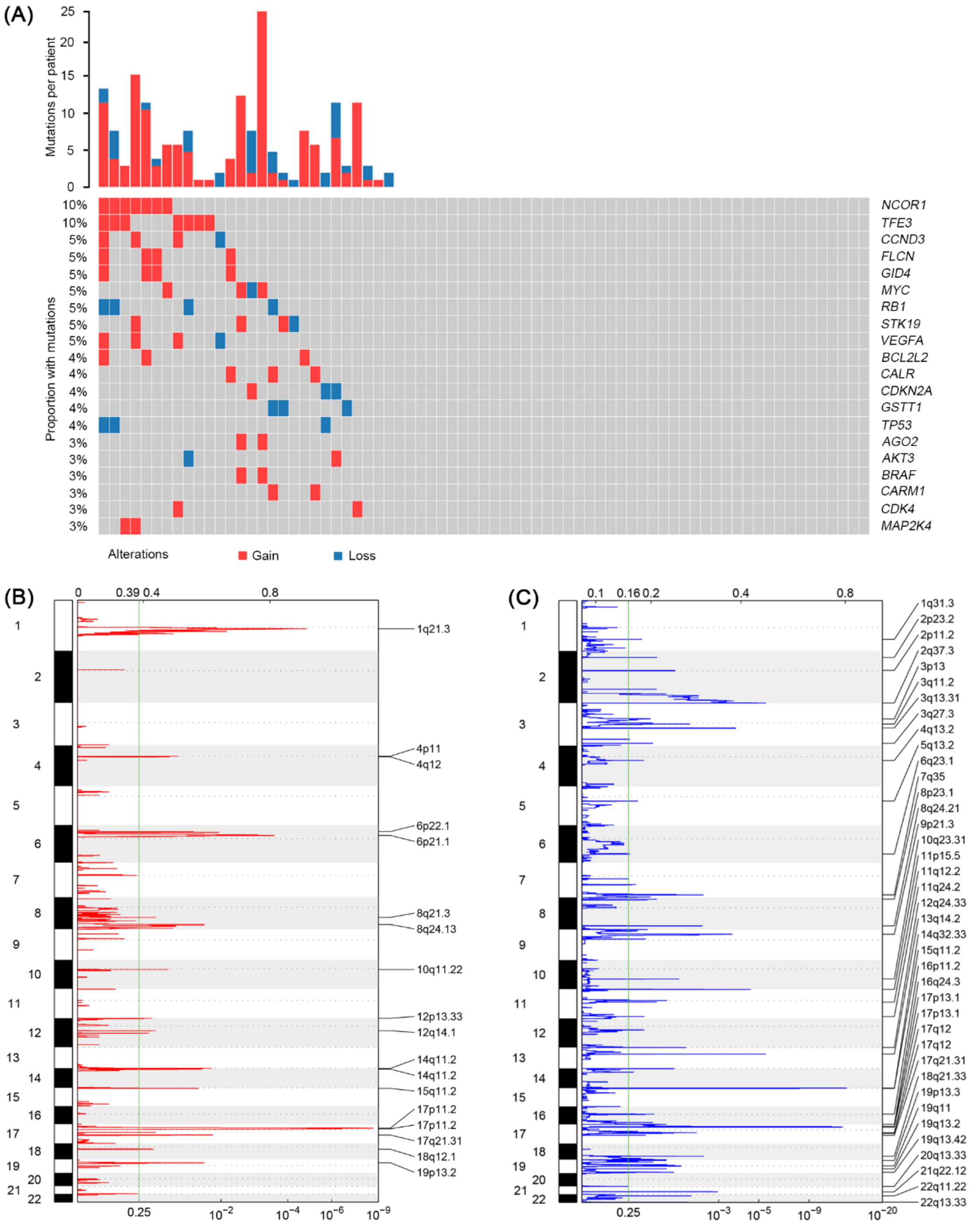

Fig. S1. The CNV landscape in Chinese patients with OS.

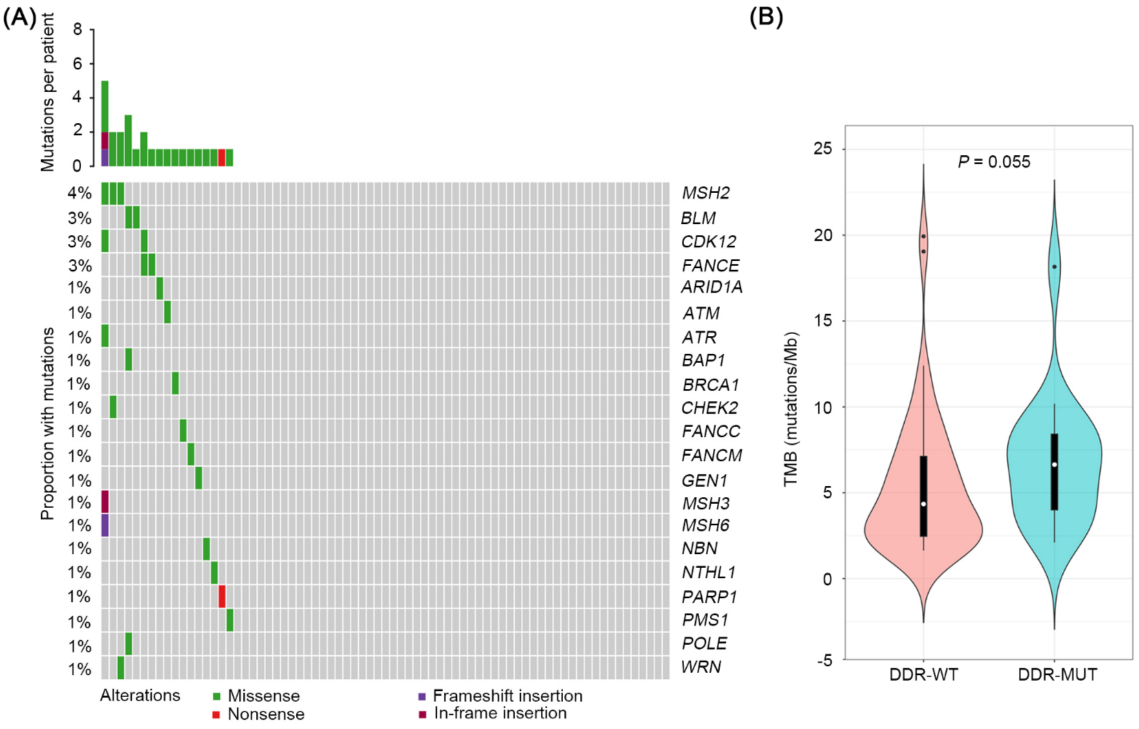

Fig. S2. DDR mutational profile and TMB analysis in Chinese OS.

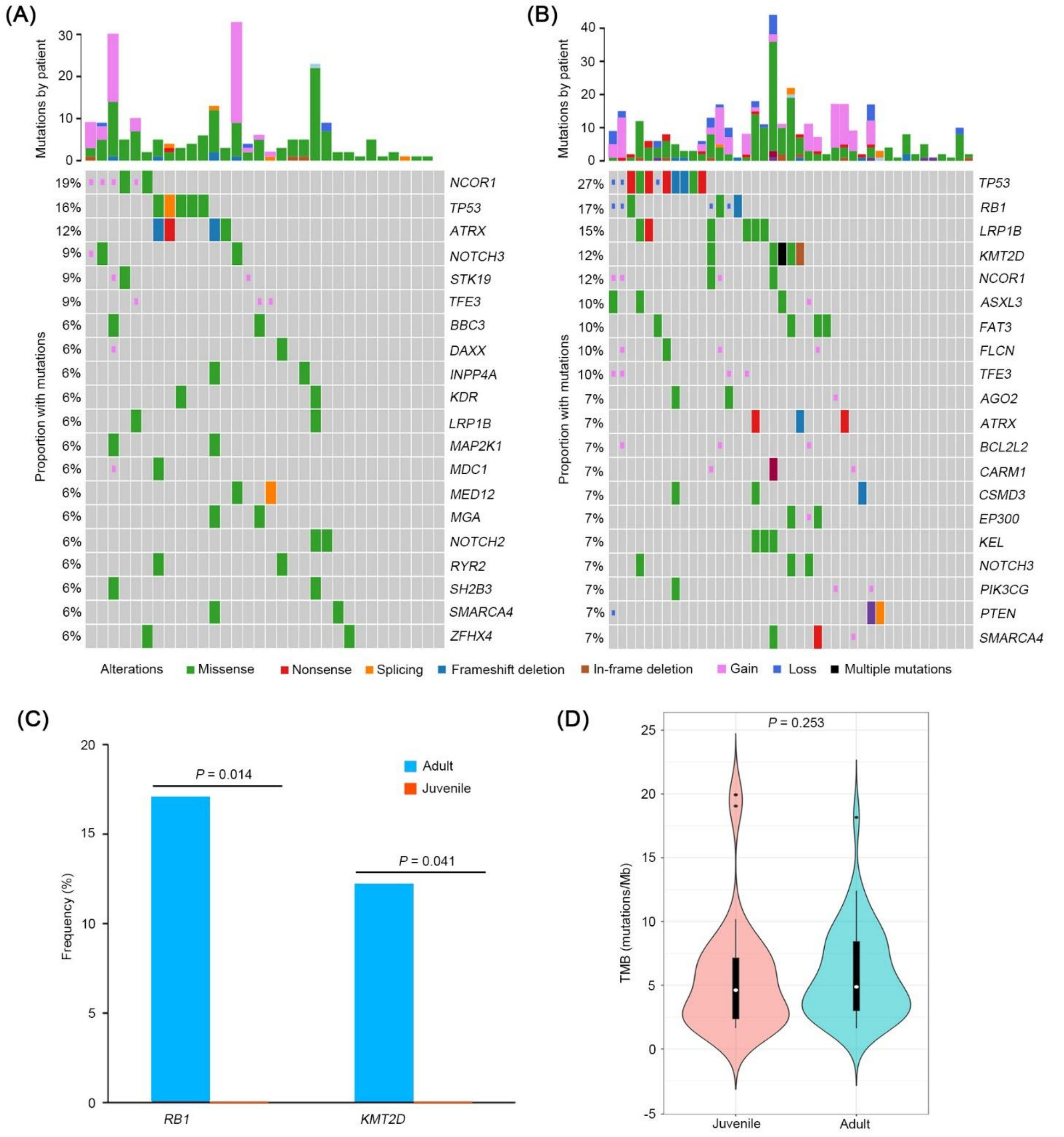

Fig. S3. The comparison of the genomic landscape between juveniles (children/adolescents) and adults.

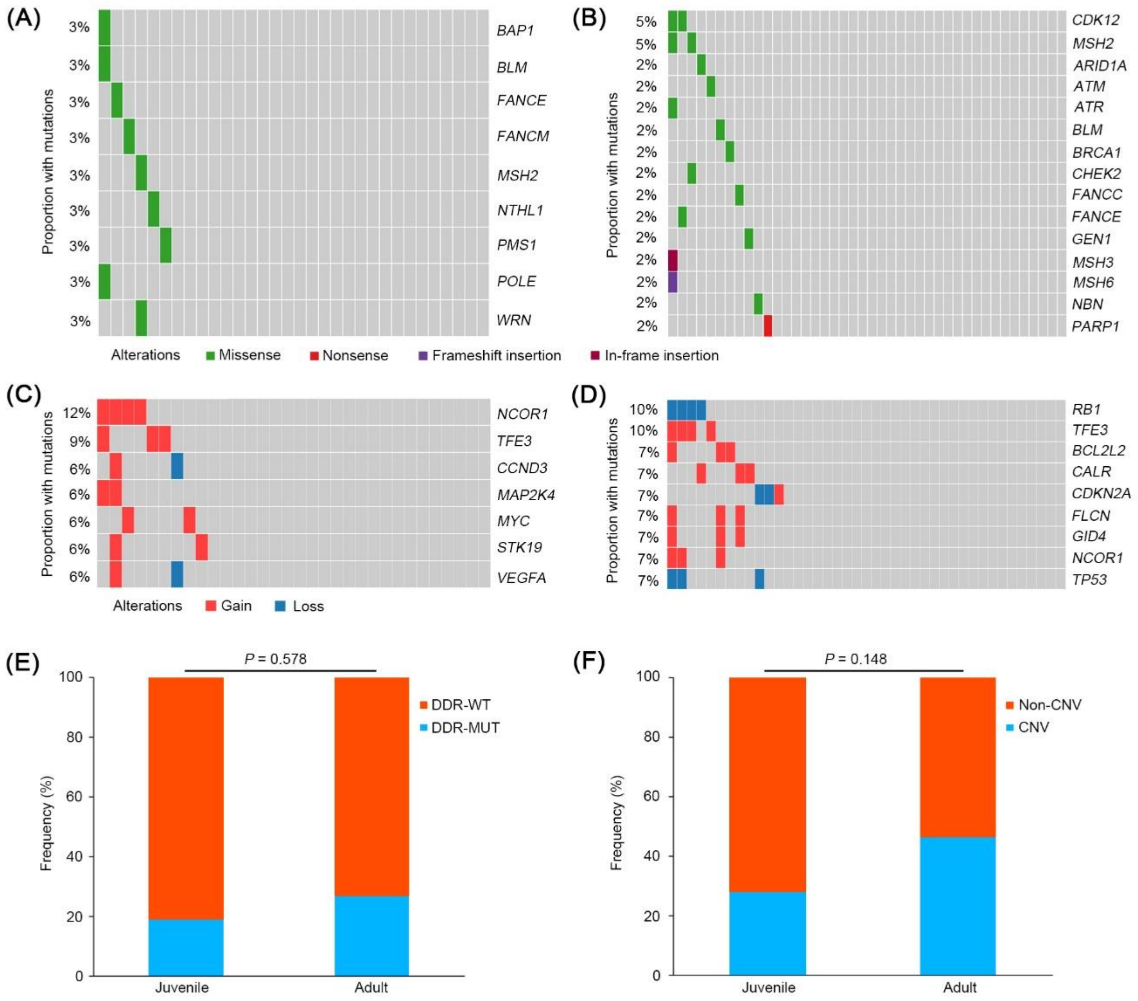

Fig. S4. The comparison of DDR mutational profile and CNV profile between juveniles and adults.

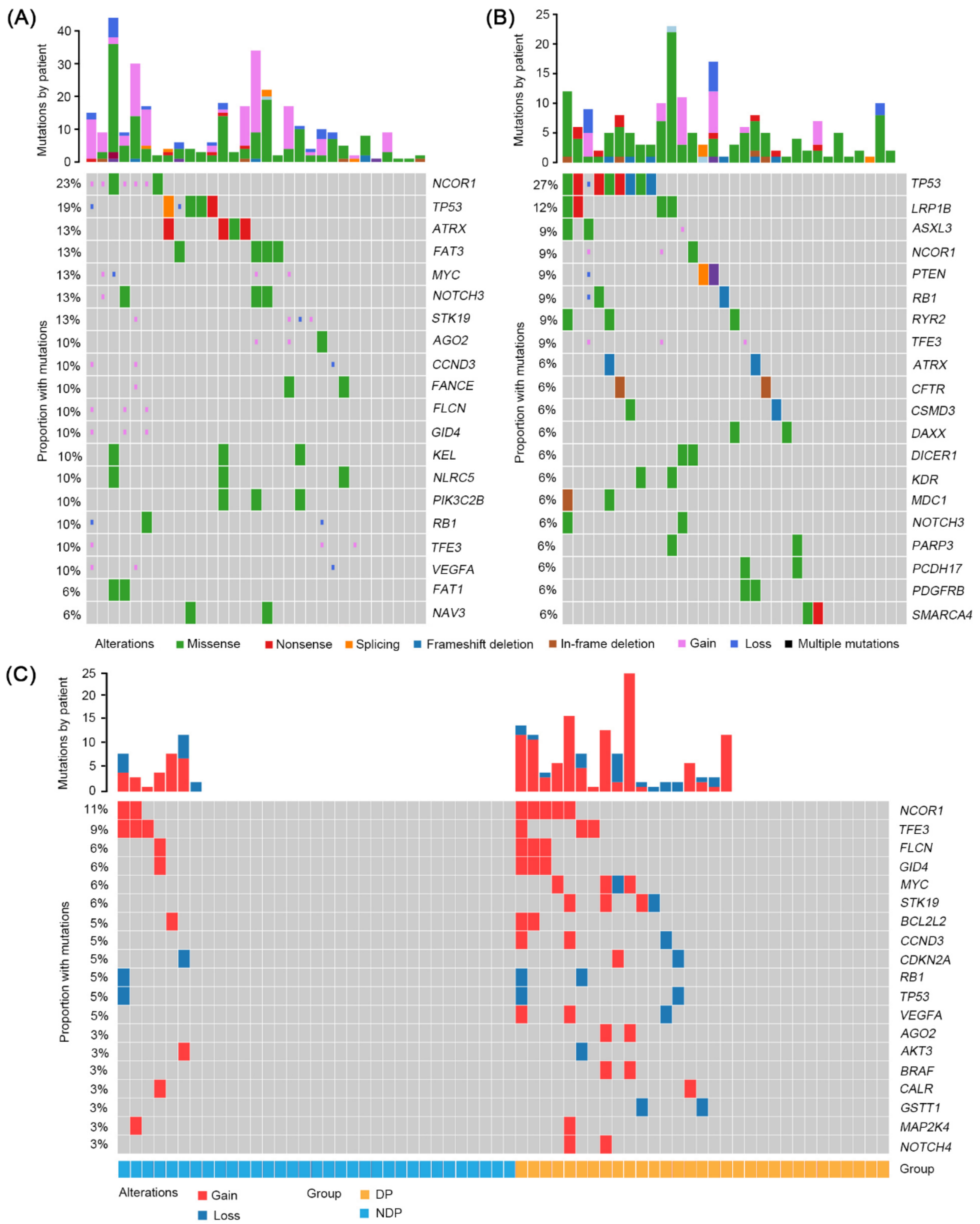

Fig. S5. The comparison of the genomic profiles between patients with and without disease progression.

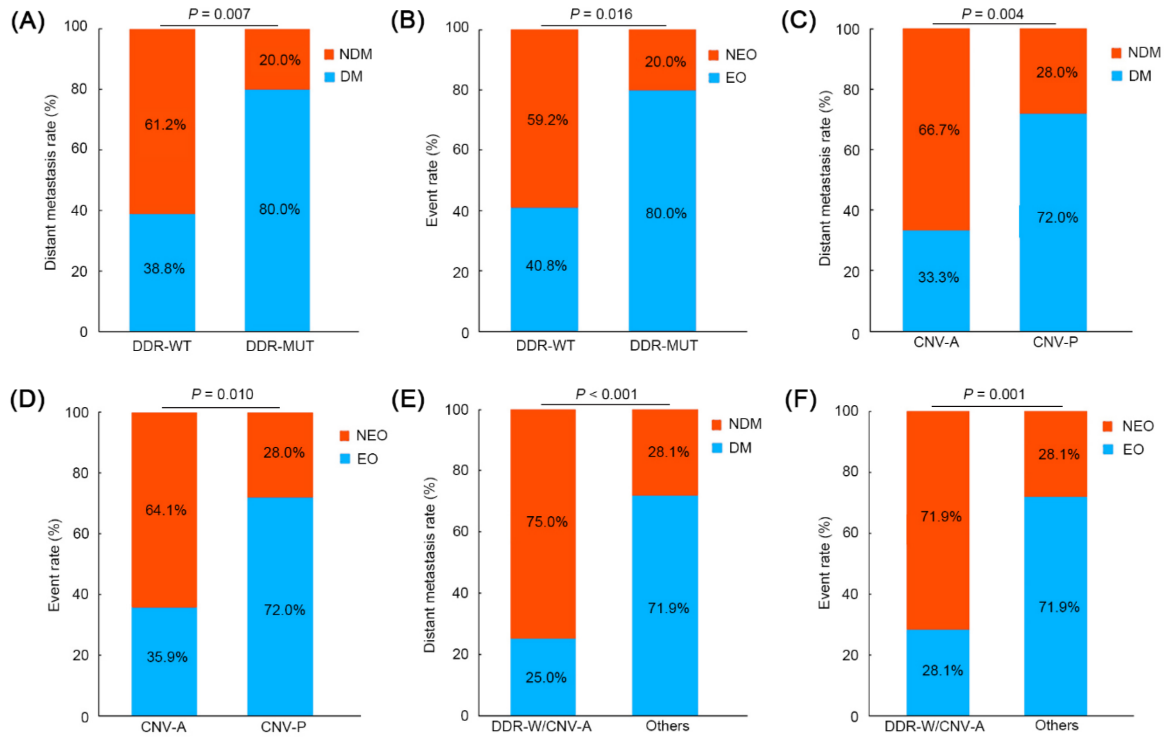

Fig. S6. The correlation of DDR mutations and CNV status with distant metastasis rate and event rate.

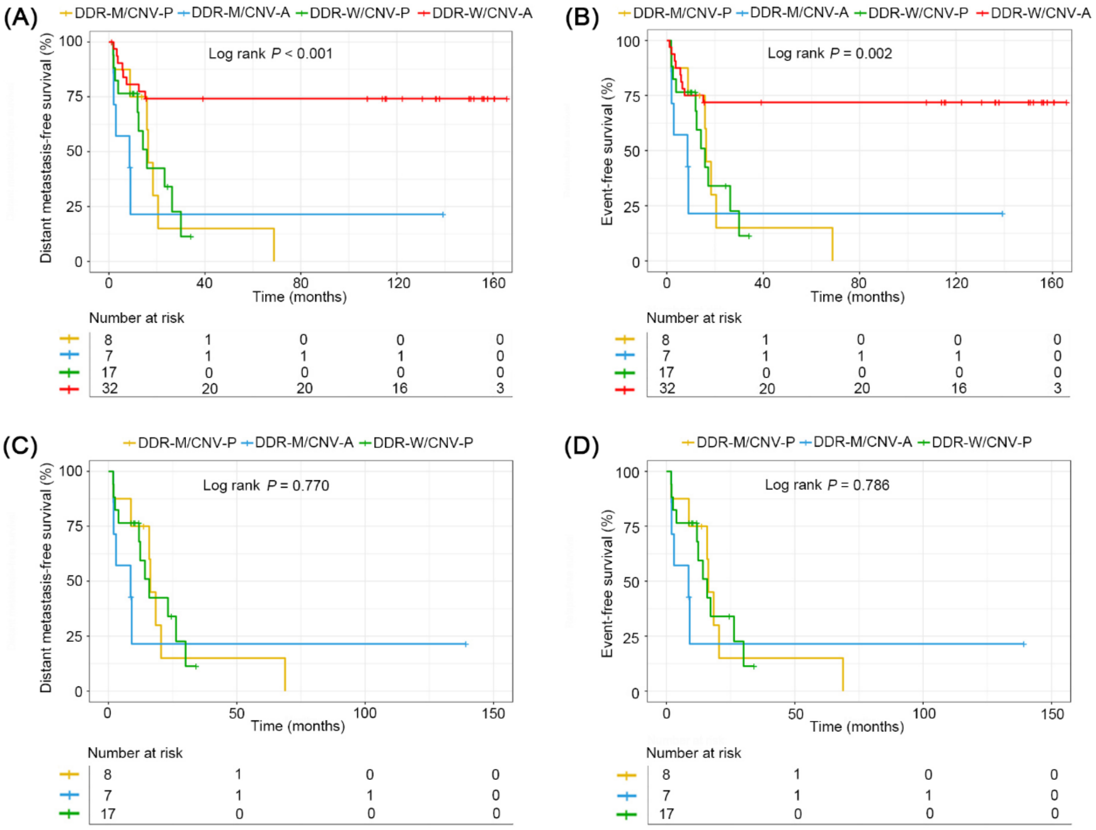

Fig. S7. Effect of a combination of DDR mutations and CNVs on DMFS and EFS.

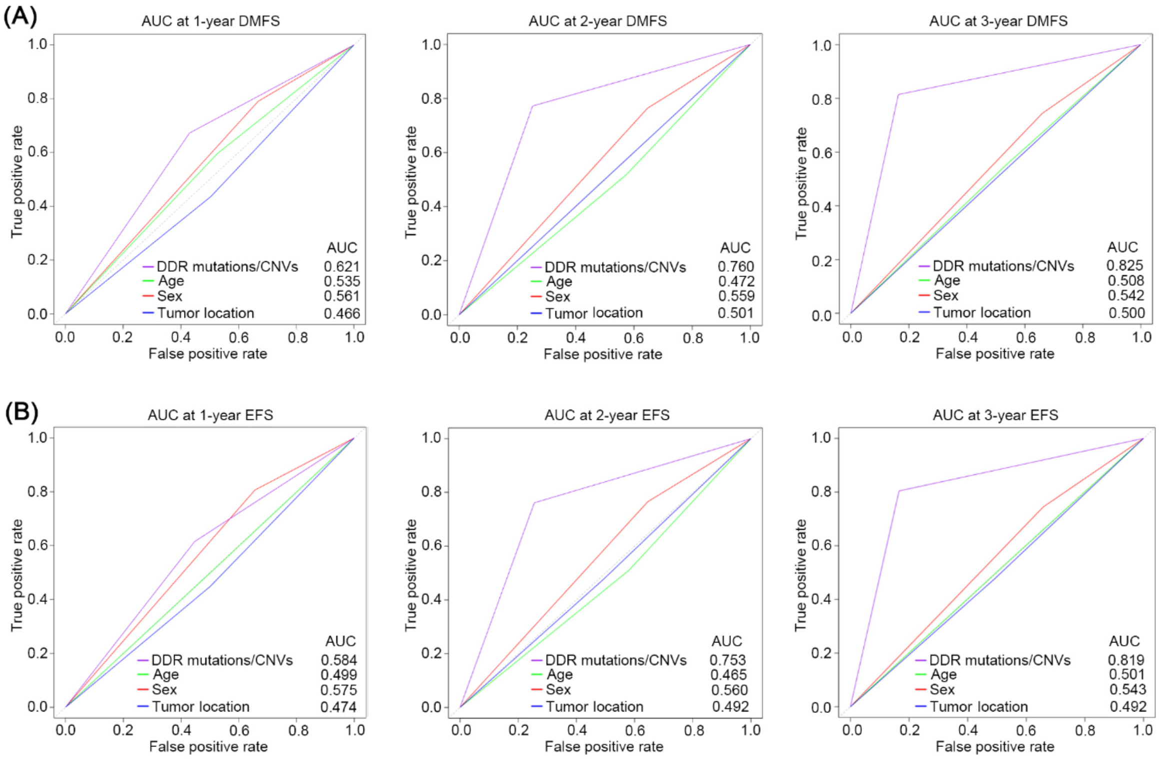

Fig. S8. Time-dependent ROC analysis of DMFS and EFS of different clinical and genomic features.

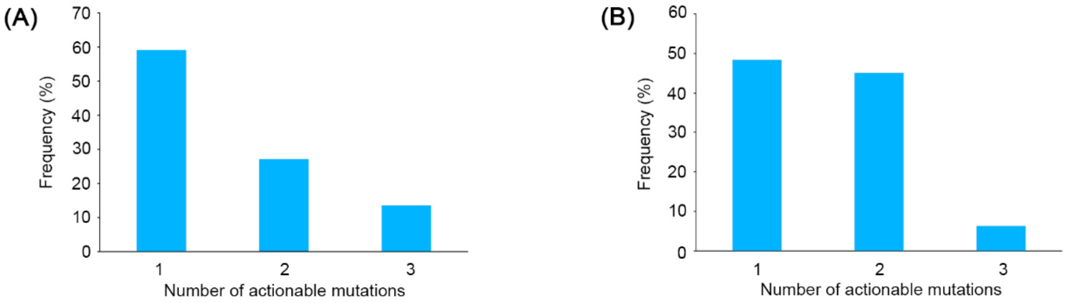

Fig. S9. Frequency of cases with clinically actionable mutations in different OS cohorts.

Table S1. Gene lists and the corresponding targeted regions in the 808 cancer-related gene panel.

No.	Gene	Targeted regions
1	*ABCB1*	Exon 12, 13, 22, 27
2	*ABCC1*	Exon 2
3	*ABCC2*	Exon 1
4	*ABCC3*	Exon 1
5	*ABCC6*	Exon 31
6	*ABCC9*	Exon 38
7	*ABCG2*	Exon 5
8	*ABL1*	Complete coding regions
9	*ABL2*	Complete coding regions
10	*ACTB*	Exon 6
11	*ACTG1*	Exon 6
12	*ACVR1*	Complete coding regions
13	*ACVR1B*	Complete coding regions
14	*ACVR2A*	Exon 3, 6, 10
15	*ADAMTS12*	Exon 24
16	*ADGRA2*	Complete coding regions
17	*ADGRG4*	Complete coding regions
18	*ADH1C*	Exon 8
19	*AGO2*	Complete coding regions
20	*AKT1*	Complete coding regions
21	*AKT2*	Complete coding regions
22	*AKT3*	Complete coding regions
23	*ALDOC*	Exon 9
24	*ALK*	Complete coding regions
25	*ALOX12B*	Complete coding regions
26	*AMER1*	Complete coding regions
27	*AMOT*	Exon 11
28	*ANK2*	Exon 2
29	*ANKRD11*	Complete coding regions
30	*APC*	Complete coding regions
31	*APCDD1*	Exon 1
32	*APOB*	Complete coding regions
33	*APOE*	Exon 1
34	*AR*	Complete coding regions
35	*ARAF*	Complete coding regions
36	*ARFRP1*	Complete coding regions
37	*ARID1A*	Complete coding regions
38	*ARID1B*	Complete coding regions
39	*ARID2*	Complete coding regions

40	*ARID5B*	Complete coding regions
41	*ASB18*	Exon 6
42	*ASTN1*	Exon 23
43	*ASTN2*	Exon 22
44	*ASXL1*	Complete coding regions
45	*ASXL2*	Complete coding regions
46	*ASXL3*	Complete coding regions
47	*ATIC*	Exon 14
48	*ATM*	Complete coding regions
49	*ATP7A*	Exon 2
50	*ATR*	Complete coding regions
51	*ATRIP*	Exon 1
52	*ATRX*	Complete coding regions
53	*AURKA*	Complete coding regions
54	*AURKB*	Complete coding regions
55	*AXIN1*	Complete coding regions
56	*AXIN2*	Complete coding regions
57	*AXL*	Complete coding regions
58	*B2M*	Complete coding regions
59	*BABAM1*	Complete coding regions
60	*BACH1*	Exon 2
61	*BAP1*	Complete coding regions
62	*BARD1*	Complete coding regions
63	*BBC3*	Complete coding regions
64	*BCL10*	Complete coding regions
65	*BCL2*	Complete coding regions
66	*BCL2L1*	Complete coding regions
67	*BCL2L11*	Complete coding regions
68	*BCL2L2*	Complete coding regions
69	*BCL6*	Complete coding regions
70	*BCOR*	Complete coding regions
71	*BCORL1*	Complete coding regions
72	*BCR*	Complete coding regions
73	*BIRC3*	Complete coding regions
74	*BLM*	Complete coding regions
75	*BMPR1A*	Complete coding regions
76	*BRAF*	Complete coding regions
77	*BRCA1*	Complete coding regions
78	*BRCA2*	Complete coding regions
79	*BRD4*	Complete coding regions
80	*BRINP3*	Complete coding regions

81	*BRIP1*	Complete coding regions
82	*BTG1*	Complete coding regions
83	*BTK*	Complete coding regions
84	*C14orf177*	Exon 3
85	*C6orf118*	Exon 9
86	*CA10*	Exon 10
87	*CALR*	Complete coding regions
88	*CARD11*	Complete coding regions
89	*CARM1*	Complete coding regions
90	*CASP7*	Exon 8
91	*CASP8*	Complete coding regions
92	*CBFB*	Complete coding regions
93	*CBL*	Complete coding regions
94	*CBR1*	Exon 1
95	*CBR3*	Exon 3
96	*CCDC6*	Complete coding regions
97	*CCND1*	Complete coding regions
98	*CCND2*	Complete coding regions
99	*CCND3*	Complete coding regions
100	*CCNE1*	Complete coding regions
101	*CD274*	Complete coding regions
102	*CD276*	Complete coding regions
103	*CD74*	Exon 1, 5, 6, 8
104	*CD79A*	Complete coding regions
105	*CD79B*	Complete coding regions
106	*CDA*	Exon 1, 2
107	*CDC42*	Complete coding regions
108	*CDC73*	Complete coding regions
109	*CDH1*	Complete coding regions
110	*CDH10*	Exon 12
111	*CDH12*	Exon 15
112	*CDH18*	Exon 15
113	*CDH9*	Exon 12
114	*CDK12*	Complete coding regions
115	*CDK4*	Complete coding regions
116	*CDK6*	Complete coding regions
117	*CDK8*	Complete coding regions
118	*CDKN1A*	Complete coding regions
119	*CDKN1B*	Complete coding regions
120	*CDKN2A*	Complete coding regions
121	*CDKN2B*	Complete coding regions

122	*CDKN2C*	Complete coding regions
123	*CEBPA*	Complete coding regions
124	*CENPA*	Complete coding regions
125	*CFTR*	Complete coding regions
126	*CHD2*	Complete coding regions
127	*CHD4*	Complete coding regions
128	*CHEK1*	Complete coding regions
129	*CHEK2*	Complete coding regions
130	*CHRM2*	Complete coding regions
131	*CHST3*	Exon 3
132	*CHUK*	Exon 21
133	*CIC*	Complete coding regions
134	*CNTNAP2*	Exon 1
135	*CNTNAP5*	Exon 1
136	*COL22A1*	Exon 65
137	*CRBN*	Exon 11
138	*CREBBP*	Complete coding regions
139	*CRKL*	Complete coding regions
140	*CRLF2*	Complete coding regions
141	*CSDE1*	Complete coding regions
142	*CSF1R*	Complete coding regions
143	*CSF3R*	Complete coding regions
144	*CSMD3*	Exon 71
145	*CTCF*	Complete coding regions
146	*CTLA4*	Complete coding regions
147	*CTNNA1*	Complete coding regions
148	*CTNNA2*	Exon 1
149	*CTNNB1*	Complete coding regions
150	*CUL3*	Complete coding regions
151	*CUL4A*	Exon 6, 8, 12, 20
152	*CUL4B*	Exon 22
153	*CXCR4*	Complete coding regions
154	*CYBA*	Exon 6
155	*CYLD*	Complete coding regions
156	*CYP17A1*	Exon 1, 4, 8
157	*CYP2B6*	Exon 1
158	*CYP2C19*	Exon 1
159	*CYP2C8*	Exon 8
160	*CYP2C9*	Exon 1
161	*CYP2D6*	Complete coding regions
162	*CYP2E1*	Exon 8

163	*CYP3A4*	Exon 10
164	*CYSLTR2*	Exon 6
165	*DAXX*	Complete coding regions
166	*DCAF12L1*	Complete coding regions
167	*DCAF12L2*	Complete coding regions
168	*DCAF4L2*	Complete coding regions
169	*DCK*	Exon 1
170	*DCLRE1C*	Exon 14
171	*DCUN1D1*	Complete coding regions
172	*DDB1*	Exon 27
173	*DDR2*	Complete coding regions
174	*DICER1*	Complete coding regions
175	*DIS3*	Complete coding regions
176	*DMD*	Exon 79
177	*DNAJB1*	Complete coding regions
178	*DNMT1*	Complete coding regions
179	*DNMT3A*	Complete coding regions
180	*DNMT3B*	Complete coding regions
181	*DOT1L*	Complete coding regions
182	*DPYD*	Exon 6, 13, 14, 22
183	*DROSHA*	Complete coding regions
184	*DUSP27*	Complete coding regions
185	*DUSP4*	Complete coding regions
186	*E2F3*	Complete coding regions
187	*EED*	Complete coding regions
188	*EGF*	Exon 1
189	*EGFL7*	Complete coding regions
190	*EGFR*	Complete coding regions
191	*EIF1AX*	Complete coding regions
192	*EIF4A2*	Complete coding regions
193	*EIF4E*	Complete coding regions
194	*ELF3*	Complete coding regions
195	*EML4*	Complete coding regions
196	*EMSY*	Complete coding regions
197	*ENG*	Complete coding regions
198	*EP300*	Complete coding regions
199	*EPAS1*	Complete coding regions
200	*EPCAM*	Complete coding regions
201	*EPHA2*	Complete coding regions
202	*EPHA3*	Complete coding regions
203	*EPHA5*	Complete coding regions

204	*EPHA7*	Complete coding regions
205	*EPHB1*	Complete coding regions
206	*EPHX1*	Exon 2
207	*ERBB2*	Complete coding regions
208	*ERBB3*	Complete coding regions
209	*ERBB4*	Complete coding regions
210	*ERCC1*	Exon 4, 10
211	*ERCC2*	Complete coding regions
212	*ERCC3*	Complete coding regions
213	*ERCC4*	Complete coding regions
214	*ERCC5*	Complete coding regions
215	*ERF*	Complete coding regions
216	*ERG*	Complete coding regions
217	*ERICH3*	Complete coding regions
218	*ERRFI1*	Complete coding regions
219	*ESR1*	Complete coding regions
220	*ESR2*	Exon 14
221	*ETV1*	Complete coding regions
222	*ETV4*	Complete coding regions
223	*ETV5*	Complete coding regions
224	*ETV6*	Complete coding regions
225	*EWSR1*	Complete coding regions
226	*EZH1*	Complete coding regions
227	*EZH2*	Complete coding regions
228	*EZR*	Exon 9-11
229	*F3*	Exon 6
230	*FAM135B*	Complete coding regions
231	*FAM175A*	Complete coding regions
232	*FAM46C*	Exon 2
233	*FAM58A*	Complete coding regions
234	*FANCA*	Complete coding regions
235	*FANCB*	Complete coding regions
236	*FANCC*	Complete coding regions
237	*FANCD2*	Complete coding regions
238	*FANCE*	Complete coding regions
239	*FANCF*	Complete coding regions
240	*FANCG*	Complete coding regions
241	*FANCI*	Exon 7, 15, 18, 25, 28, 32, 33, 34, 36, 37
242	*FANCL*	Complete coding regions
243	*FANCM*	Exon 8, 13, 14
244	*FAS*	Complete coding regions

245	*FAT1*	Complete coding regions
246	*FAT3*	Complete coding regions
247	*FBN2*	Exon 65
248	*FBXL7*	Exon 1
249	*FBXW7*	Complete coding regions
250	*FCGR3A*	Exon 5
251	*FES*	Exon 2
252	*FGD1*	Exon 18
253	*FGF10*	Complete coding regions
254	*FGF12*	Exon 6
255	*FGF14*	Complete coding regions
256	*FGF19*	Complete coding regions
257	*FGF23*	Complete coding regions
258	*FGF3*	Complete coding regions
259	*FGF4*	Complete coding regions
260	*FGF6*	Complete coding regions
261	*FGF7*	Exon 2
262	*FGFR1*	Complete coding regions
263	*FGFR2*	Complete coding regions
264	*FGFR3*	Complete coding regions
265	*FGFR4*	Complete coding regions
266	*FH*	Complete coding regions
267	*FIP1L1*	Exon 1
268	*FLCN*	Complete coding regions
269	*FLT1*	Complete coding regions
270	*FLT3*	Complete coding regions
271	*FLT4*	Complete coding regions
272	*FOXA1*	Complete coding regions
273	*FOXL2*	Complete coding regions
274	*FOXO1*	Complete coding regions
275	*FOXP1*	Complete coding regions
276	*FRK*	Exon 6
277	*FRS2*	Complete coding regions
278	*FRYL*	Exon 64
279	*FUBP1*	Complete coding regions
280	*FYN*	Complete coding regions
281	*G6PC3*	Exon 1
282	*GABRA2*	Exon 10
283	*GABRA6*	Complete coding regions
284	*GALNT12*	Complete coding regions
285	*GAPDH*	Exon 2

286	*GAST*	Exon 2
287	*GATA1*	Complete coding regions
288	*GATA2*	Complete coding regions
289	*GATA3*	Complete coding regions
290	*GATA4*	Complete coding regions
291	*GATA6*	Complete coding regions
292	*GEN1*	Complete coding regions
293	*GGH*	Exon 9
294	*GID4*	Complete coding regions
295	*GLI1*	Complete coding regions
296	*GNA11*	Complete coding regions
297	*GNA13*	Complete coding regions
298	*GNAQ*	Complete coding regions
299	*GNAS*	Complete coding regions
300	*GOPC*	Complete coding regions
301	*GPC3*	Complete coding regions
302	*GPR158*	Exon 1
303	*GPS2*	Complete coding regions
304	*GREM1*	Complete coding regions
305	*GRIK3*	Exon 16
306	*GRIN2A*	Complete coding regions
307	*GRM3*	Complete coding regions
308	*GRM8*	Exon 10
309	*GSK3B*	Complete coding regions
310	*GSTA1*	Exon 7
311	*GSTM1*	Complete coding regions
312	*GSTM3*	Exon 9
313	*GSTP1*	Exon 5, 6
314	*GSTT1*	Complete coding regions
315	*H3F3A*	Complete coding regions
316	*H3F3AP4*	Complete coding regions
317	*H3F3B*	Complete coding regions
318	*H3F3C*	Complete coding regions
319	*HAPLN1*	Exon 5
320	*HCN1*	Exon 8
321	*HDAC9*	Exon 2
322	*HGF*	Complete coding regions
323	*HIST1H1C*	Complete coding regions
324	*HIST1H2BD*	Complete coding regions
325	*HIST1H3A*	Complete coding regions
326	*HIST1H3B*	Complete coding regions

327	*HIST1H3C*	Complete coding regions
328	*HIST1H3D*	Complete coding regions
329	*HIST1H3E*	Complete coding regions
330	*HIST1H3F*	Complete coding regions
331	*HIST1H3G*	Complete coding regions
332	*HIST1H3H*	Complete coding regions
333	*HIST1H3I*	Complete coding regions
334	*HIST1H3J*	Complete coding regions
335	*HIST2H3A*	Complete coding regions
336	*HIST2H3C*	Complete coding regions
337	*HIST2H3D*	Complete coding regions
338	*HIST3H3*	Complete coding regions
339	*HLA-A*	Complete coding regions
340	*HLA-B*	Complete coding regions
341	*HLA-C*	Complete coding regions
342	*HNF1A*	Complete coding regions
343	*HOXB13*	Complete coding regions
344	*HRAS*	Complete coding regions
345	*HSD3B1*	Complete coding regions
346	*HSP90AA1*	Complete coding regions
347	*HTR1A*	Complete coding regions
348	*ICOSLG*	Complete coding regions
349	*ID3*	Complete coding regions
350	*IDH1*	Complete coding regions
351	*IDH2*	Complete coding regions
352	*IFNGR1*	Complete coding regions
353	*IFNL3*	Exon 3
354	*IGF1*	Complete coding regions
355	*IGF1R*	Complete coding regions
356	*IGF2*	Complete coding regions
357	*IGFL3*	Exon 4
358	*IKBKE*	Complete coding regions
359	*IKZF1*	Complete coding regions
360	*IL10*	Complete coding regions
361	*IL7R*	Complete coding regions
362	*INHA*	Complete coding regions
363	*INHBA*	Complete coding regions
364	*INPP4A*	Complete coding regions
365	*INPP4B*	Complete coding regions
366	*INPPL1*	Complete coding regions
367	*INSR*	Complete coding regions

368	*INSRR*	Exon 22
369	*IQCJ*	Exon 1
370	*IRF2*	Complete coding regions
371	*IRF4*	Complete coding regions
372	*IRS1*	Complete coding regions
373	*IRS2*	Complete coding regions
374	*ITPA*	Exon 1
375	*JAK1*	Complete coding regions
376	*JAK2*	Complete coding regions
377	*JAK3*	Complete coding regions
378	*JUN*	Exon 1
379	*KAT6A*	Complete coding regions
380	*KCNA4*	Complete coding regions
381	*KCND2*	Complete coding regions
382	*KCNJ3*	Exon 1
383	*KCNT2*	Exon 25
384	*KDM5A*	Complete coding regions
385	*KDM5C*	Complete coding regions
386	*KDM6A*	Complete coding regions
387	*KDR*	Complete coding regions
388	*KEAP1*	Complete coding regions
389	*KEL*	Complete coding regions
390	*KIF2B*	Complete coding regions
391	*KIF5B*	Exon 15
392	*KIT*	Complete coding regions
393	*KLC1*	Exon 2
394	*KLF4*	Complete coding regions
395	*KLHL1*	Exon 11
396	*KLHL6*	Complete coding regions
397	*KMT2A*	Complete coding regions
398	*KMT2B*	Complete coding regions
399	*KMT2C*	Complete coding regions
400	*KMT2D*	Complete coding regions
401	*KMT5A*	Complete coding regions
402	*KNSTRN*	Complete coding regions
403	*KRAS*	Complete coding regions
404	*LATS1*	Complete coding regions
405	*LATS2*	Complete coding regions
406	*LEPR*	Exon 3
407	*LIG3*	Exon 2, 20
408	*LIG4*	Exon 3

409	*LMO1*	Complete coding regions
410	*LOC349160*	Exon 2
411	*LPL*	Exon 1
412	*LPPR4*	Complete coding regions
413	*LRFN5*	Complete coding regions
414	*LRIG3*	Exon 19
415	*LRP1B*	Complete coding regions
416	*LRRC4C*	Complete coding regions
417	*LRRIQ3*	Exon 8
418	*LRRK2*	Exon 1
419	*LRRTM4*	Complete coding regions
420	*LTK*	Exon 14
421	*LYN*	Complete coding regions
422	*LZTR1*	Complete coding regions
423	*MAD1L1*	Exon 19
424	*MAGI2*	Complete coding regions
425	*MALT1*	Complete coding regions
426	*MAP2K1*	Complete coding regions
427	*MAP2K2*	Complete coding regions
428	*MAP2K4*	Complete coding regions
429	*MAP3K1*	Complete coding regions
430	*MAP3K13*	Complete coding regions
431	*MAP3K14*	Complete coding regions
432	*MAP4K3*	Exon 14
433	*MAPK1*	Complete coding regions
434	*MAPK3*	Complete coding regions
435	*MAPKAP1*	Complete coding regions
436	*MAX*	Complete coding regions
437	*MBD4*	Exon 8
438	*MCL1*	Complete coding regions
439	*MDC1*	Complete coding regions
440	*MDH2*	Complete coding regions
441	*MDM2*	Complete coding regions
442	*MDM4*	Complete coding regions
443	*MED12*	Complete coding regions
444	*MEF2B*	Complete coding regions
445	*MEN1*	Complete coding regions
446	*MET*	Complete coding regions
447	*MGA*	Complete coding regions
448	*MITF*	Complete coding regions
449	*MKRN3*	Complete coding regions

450	*MLH1*	Complete coding regions
451	*MLH3*	Exon 2, 7
452	*MNAT1*	Exon 1
453	*MOCS2*	Exon 7
454	*MPL*	Complete coding regions
455	*MRE11A*	Complete coding regions
456	*MS4A3*	Exon 2
457	*MSH2*	Complete coding regions
458	*MSH3*	Complete coding regions
459	*MSH6*	Complete coding regions
460	*MSI1*	Complete coding regions
461	*MSI2*	Complete coding regions
462	*MST1*	Complete coding regions
463	*MST1R*	Complete coding regions
464	*MTHFD1*	Exon 1
465	*MTHFR*	Exon 5
466	*MTOR*	Complete coding regions
467	*MUTYH*	Complete coding regions
468	*MYB*	Complete coding regions
469	*MYC*	Complete coding regions
470	*MYCL1*	Complete coding regions
471	*MYCN*	Complete coding regions
472	*MYD88*	Complete coding regions
473	*MYOD1*	Complete coding regions
474	*NAT2*	Exon 2
475	*NAV3*	Exon 1
476	*NBN*	Complete coding regions
477	*NCAM1*	Exon 1, 2
478	*NCOA3*	Complete coding regions
479	*NCOA4*	Complete coding regions
480	*NCOR1*	Complete coding regions
481	*NEGR1*	Complete coding regions
482	*NEIL1*	Exon 3
483	*NEIL3*	Exon 1
484	*NF1*	Complete coding regions
485	*NF2*	Complete coding regions
486	*NFE2L2*	Complete coding regions
487	*NFKBIA*	Complete coding regions
488	*NKX2-1*	Complete coding regions
489	*NKX3-1*	Complete coding regions
490	*NLRC5*	Complete coding regions

491	*NLRP3*	Complete coding regions
492	*NLRP5*	Exon 1
493	*NOTCH1*	Complete coding regions
494	*NOTCH2*	Complete coding regions
495	*NOTCH3*	Complete coding regions
496	*NOTCH4*	Complete coding regions
497	*NPM1*	Complete coding regions
498	*NQO1*	Exon 6
499	*NRAS*	Complete coding regions
500	*NRG1*	Complete coding regions
501	*NSD1*	Complete coding regions
502	*NTHL1*	Complete coding regions
503	*NTRK1*	Complete coding regions
504	*NTRK2*	Complete coding regions
505	*NTRK3*	Complete coding regions
506	*NUF2*	Complete coding regions
507	*NUP93*	Complete coding regions
508	*NUTM1*	Complete coding regions
509	*PAK1*	Complete coding regions
510	*PAK3*	Complete coding regions
511	*PAK6*	Complete coding regions
512	*PAK7*	Complete coding regions
513	*PALB2*	Complete coding regions
514	*PALLD*	Complete coding regions
515	*PAPPA2*	Exon 2
516	*PARK2*	Complete coding regions
517	*PARP1*	Complete coding regions
518	*PARP2*	Exon 9, 13
519	*PARP3*	Exon 4, 10
520	*PARP4*	Complete coding regions
521	*PAX5*	Complete coding regions
522	*PAX8*	Complete coding regions
523	*PBRM1*	Complete coding regions
524	*PCDH10*	Complete coding regions
525	*PCDH17*	Complete coding regions
526	*PDCD1*	Complete coding regions
527	*PDCD1LG2*	Complete coding regions
528	*PDGFRA*	Complete coding regions
529	*PDGFRB*	Complete coding regions
530	*PDHA2*	Complete coding regions
531	*PDK1*	Complete coding regions

532	*PDPK1*	Complete coding regions
533	*PER1*	Exon 23
534	*PGR*	Complete coding regions
535	*PHOX2B*	Complete coding regions
536	*PIK3C2B*	Complete coding regions
537	*PIK3C2G*	Complete coding regions
538	*PIK3C3*	Complete coding regions
539	*PIK3CA*	Complete coding regions
540	*PIK3CB*	Complete coding regions
541	*PIK3CD*	Complete coding regions
542	*PIK3CG*	Complete coding regions
543	*PIK3R1*	Complete coding regions
544	*PIK3R2*	Complete coding regions
545	*PIK3R3*	Complete coding regions
546	*PIM1*	Complete coding regions
547	*PIP5K1A*	Exon 1
548	*PKD1*	Complete coding regions
549	*PLCG2*	Complete coding regions
550	*PLK2*	Complete coding regions
551	*PMAIP1*	Complete coding regions
552	*PML*	Complete coding regions
553	*PMS1*	Complete coding regions
554	*PMS2*	Complete coding regions
555	*PNRC1*	Complete coding regions
556	*POLD1*	Complete coding regions
557	*POLDIP2*	Exon 11
558	*POLE*	Complete coding regions
559	*POLM*	Exon 9
560	*POM121L12*	Complete coding regions
561	*PPARD*	Exon 2
562	*PPARG*	Complete coding regions
563	*PPM1D*	Complete coding regions
564	*PPP2R1A*	Complete coding regions
565	*PPP4R2*	Complete coding regions
566	*PPP6C*	Complete coding regions
567	*PRCC*	Complete coding regions
568	*PRDM1*	Complete coding regions
569	*PRDM14*	Complete coding regions
570	*PREX2*	Complete coding regions
571	*PRIM2*	Exon 2
572	*PRKACA*	Exon 10

573	*PRKAR1A*	Complete coding regions
574	*PRKCI*	Complete coding regions
575	*PRKD1*	Complete coding regions
576	*PRKDC*	Complete coding regions
577	*PRSS1*	Complete coding regions
578	*PRSS8*	Complete coding regions
579	*PTCH1*	Complete coding regions
580	*PTEN*	Complete coding regions
581	*PTP4A1*	Complete coding regions
582	*PTPN11*	Complete coding regions
583	*PTPRD*	Complete coding regions
584	*PTPRS*	Complete coding regions
585	*PTPRT*	Complete coding regions
586	*PXDNL*	Complete coding regions
587	*RAB35*	Complete coding regions
588	*RAC1*	Complete coding regions
589	*RAC2*	Complete coding regions
590	*RAD21*	Complete coding regions
591	*RAD50*	Complete coding regions
592	*RAD51*	Complete coding regions
593	*RAD51B*	Complete coding regions
594	*RAD51C*	Complete coding regions
595	*RAD51D*	Complete coding regions
596	*RAD52*	Complete coding regions
597	*RAD54L*	Complete coding regions
598	*RAF1*	Complete coding regions
599	*RANBP2*	Complete coding regions
600	*RARA*	Complete coding regions
601	*RASA1*	Complete coding regions
602	*RB1*	Complete coding regions
603	*RBBP8*	Exon 11
604	*RBM10*	Complete coding regions
605	*RECQL*	Complete coding regions
606	*RECQL4*	Complete coding regions
607	*REG3A*	Exon 6
608	*REL*	Complete coding regions
609	*RET*	Complete coding regions
610	*REV1*	Exon 23
611	*REV3L*	Exon 33
612	*RFWD2*	Complete coding regions
613	*RHBDF2*	Complete coding regions

614	*RHEB*	Complete coding regions
615	*RHOA*	Complete coding regions
616	*RICTOR*	Complete coding regions
617	*RINT1*	Complete coding regions
618	*RIT1*	Complete coding regions
619	*RNF43*	Complete coding regions
620	*ROCK1*	Complete coding regions
621	*ROS1*	Complete coding regions
622	*RP1L1*	Complete coding regions
623	*RPA1*	Exon 5, 12
624	*RPL11*	Complete coding regions
625	*RPL35A*	Complete coding regions
626	*RPL5*	Complete coding regions
627	*RPP30*	Exon 6
628	*RPS10*	Complete coding regions
629	*RPS17*	Complete coding regions
630	*RPS19*	Complete coding regions
631	*RPS24*	Complete coding regions
632	*RPS26*	Complete coding regions
633	*RPS6KA4*	Complete coding regions
634	*RPS6KB2*	Complete coding regions
635	*RPS7*	Complete coding regions
636	*RPTOR*	Complete coding regions
637	*RRAGC*	Complete coding regions
638	*RRAS*	Complete coding regions
639	*RRAS2*	Complete coding regions
640	*RRM1*	Exon 19
641	*RRM2*	Exon 4
642	*RTEL1*	Complete coding regions
643	*RUNX1*	Complete coding regions
644	*RUNX1T1*	Complete coding regions
645	*RXRA*	Complete coding regions
646	*RYBP*	Complete coding regions
647	*RYR2*	Exon 1
648	*SALL1*	Complete coding regions
649	*SDC4*	Complete coding regions
650	*SDHA*	Complete coding regions
651	*SDHAF2*	Complete coding regions
652	*SDHB*	Complete coding regions
653	*SDHC*	Complete coding regions
654	*SDHD*	Complete coding regions

655	*SESN1*	Complete coding regions
656	*SESN2*	Complete coding regions
657	*SESN3*	Complete coding regions
658	*SETBP1*	Complete coding regions
659	*SETD2*	Complete coding regions
660	*SETD8*	Complete coding regions
661	*SETMAR*	Complete coding regions
662	*SF3B1*	Complete coding regions
663	*SH2B3*	Complete coding regions
664	*SH2D1A*	Complete coding regions
665	*SHMT1*	Exon 12
666	*SHOC2*	Complete coding regions
667	*SHQ1*	Complete coding regions
668	*SLC14A2*	Exon 3
669	*SLC19A1*	Exon 6
670	*SLC22A2*	Exon 11
671	*SLC22A4*	Exon 1
672	*SLC34A2*	Exon 4, 5, 13
673	*SLC45A2*	Exon 7
674	*SLC8A1*	Complete coding regions
675	*SLCO1B1*	Exon 6
676	*SLCO1B3*	Exon 3
677	*SLIT2*	Complete coding regions
678	*SLIT3*	Exon 36
679	*SLITRK1*	Complete coding regions
680	*SLITRK2*	Complete coding regions
681	*SLITRK3*	Complete coding regions
682	*SLX4*	Complete coding regions
683	*SMAD2*	Complete coding regions
684	*SMAD3*	Complete coding regions
685	*SMAD4*	Complete coding regions
686	*SMAD7*	Complete coding regions
687	*SMARCA4*	Complete coding regions
688	*SMARCB1*	Complete coding regions
689	*SMARCD1*	Complete coding regions
690	*SMO*	Complete coding regions
691	*SMYD3*	Complete coding regions
692	*SOCS1*	Complete coding regions
693	*SOS1*	Complete coding regions
694	*SOX10*	Complete coding regions
695	*SOX17*	Complete coding regions

696	*SOX2*	Exon 1
697	*SOX9*	Complete coding regions
698	*SPEN*	Complete coding regions
699	*SPG7*	Exon 11
700	*SPHKAP*	Complete coding regions
701	*SPINK1*	Complete coding regions
702	*SPOP*	Complete coding regions
703	*SPRED1*	Complete coding regions
704	*SPTA1*	Complete coding regions
705	*SRC*	Complete coding regions
706	*SRSF2*	Complete coding regions
707	*ST6GAL2*	Exon 6
708	*STAG2*	Complete coding regions
709	*STAT3*	Complete coding regions
710	*STAT4*	Complete coding regions
711	*STAT5A*	Complete coding regions
712	*STAT5B*	Complete coding regions
713	*STK11*	Complete coding regions
714	*STK19*	Complete coding regions
715	*STK40*	Complete coding regions
716	*STT3A*	Exon 19
717	*SUFU*	Complete coding regions
718	*SUZ12*	Complete coding regions
719	*SYK*	Complete coding regions
720	*TAF1*	Complete coding regions
721	*TAP1*	Complete coding regions
722	*TAP2*	Complete coding regions
723	*TBL1XR1*	Complete coding regions
724	*TBP*	Exon 2
725	*TBX3*	Complete coding regions
726	*TCEB1*	Complete coding regions
727	*TCF3*	Complete coding regions
728	*TCF7L2*	Complete coding regions
729	*TDG*	Exon 1
730	*TEK*	Complete coding regions
731	*TEKT4*	Exon 1-6
732	*TERC*	Complete coding regions
733	*TERT*	Complete coding regions
734	*TET1*	Complete coding regions
735	*TET2*	Complete coding regions
736	*TFE3*	Complete coding regions

737	*TG*	Complete coding regions
738	*TGFB1*	Exon 3
739	*TGFBR1*	Complete coding regions
740	*TGFBR2*	Complete coding regions
741	*TGFBR3*	Exon 18
742	*TIPARP*	Exon 2, 6
743	*TLR4*	Complete coding regions
744	*TMEM127*	Complete coding regions
745	*TMPRSS2*	Complete coding regions
746	*TNFAIP3*	Complete coding regions
747	*TNFRSF14*	Complete coding regions
748	*TNFRSF17*	Exon 1
749	*TNN*	Complete coding regions
750	*TNR*	Complete coding regions
751	*TOP1*	Complete coding regions
752	*TOP2A*	Complete coding regions
753	*TOPBP1*	Exon 28
754	*TP53*	Complete coding regions
755	*TP53BP1*	Complete coding regions
756	*TP63*	Complete coding regions
757	*TPM3*	Exon 4
758	*TPMT*	Exon 4, 6, 9
759	*TRAF2*	Complete coding regions
760	*TRAF7*	Complete coding regions
761	*TRIM58*	Exon 1
762	*TRRAP*	Exon 2
763	*TSC1*	Complete coding regions
764	*TSC2*	Complete coding regions
765	*TSHR*	Complete coding regions
766	*TSHZ3*	Complete coding regions
767	*TYK2*	Exon 3, 14, 16, 20, 22
768	*TYMP*	Exon 10
769	*U2AF1*	Complete coding regions
770	*UGT1A1*	Exon 1
771	*UGT1A8*	Exon 1
772	*UPF1*	Complete coding regions
773	*USP1*	Exon 2
774	*VEGFA*	Complete coding regions
775	*VHL*	Complete coding regions
776	*VTCN1*	Complete coding regions
777	*WEE1*	Complete coding regions

778	*WHSC1*	Complete coding regions
779	*WHSC1L1*	Complete coding regions
780	*WISP3*	Complete coding regions
781	*WRN*	Complete coding regions
782	*WT1*	Complete coding regions
783	*WWTR1*	Complete coding regions
784	*XIAP*	Complete coding regions
785	*XPA*	Complete coding regions
786	*XPC*	Complete coding regions
787	*XPO1*	Complete coding regions
788	*XRCC1*	Exon 10
789	*XRCC2*	Complete coding regions
790	*XRCC3*	Exon 7
791	*YAP1*	Complete coding regions
792	*YES1*	Complete coding regions
793	*ZAN*	Exon 2
794	*ZBTB2*	Complete coding regions
795	*ZFHX3*	Complete coding regions
796	*ZFHX4*	Complete coding regions
797	*ZIC1*	Complete coding regions
798	*ZIC4*	Complete coding regions
799	*ZIM2*	Exon 12
800	*ZNF217*	Complete coding regions
801	*ZNF423*	Complete coding regions
802	*ZNF521*	Complete coding regions
803	*ZNF536*	Complete coding regions
804	*ZNF703*	Complete coding regions
805	*ZNF804A*	Complete coding regions
806	*ZNF804B*	Complete coding regions
807	*ZNF831*	Complete coding regions
808	*ZRSR2*	Complete coding regions

Table S2. Clinical characteristics among 73 patients with osteosarcoma.

Clinical characteristics	Number (%)
Age, years, median (range)	19 (5-64)
Sex	
Male	50 (68.5%)
Female	23 (31.5%)
Primary tumor lesions	
Femur	36 (49.3%)
Tibia	20 (27.4%)
Humerus	8 (10.9%)
Ilium	2 (2.7%)
Thigh	2 (2.7%)
Fibula	1 (1.4%)
Calcaneus	1 (1.4%)
Rib	1 (1.4%)
Tarsus	1 (1.4%)
Foot	1 (1.4%)
Stage at diagnosis	
II	64 (87.7%)
III	9 (12.3%)
Initial metastasis (n = 9)	
Lung metastasis	5 (55.6%)
Metastasis of lung and other sites	4 (44.4%)
Metastasis after surgery (n = 31)	
Lung metastasis	23 (74.2%)
Metastasis of lung and other sites	8 (25.8%)

Table S3. All the genomic alterations identified in OS patients.

Case. No	Gene	Chromosome	Start	End	Reference	Alteration	Frequency	pHGVS	Type
P1	*NAV3*	chr12	78444583	78444583	A	T	0.33	p.E724D	missense
P1	*TP53*	chr17	7578416	7578416	C	A	0.41	p.V172F	missense
P2	*ARID1A*	chr1	27106125	27106125	T	G	0.04	p.D1912E	missense
P2	*FAT3*	chr11	92086966	92086966	A	C	0.05	p.N563T	missense
P3	*NCOR1*	chr17	16042464	16042464	G	T	0.05	p.P404T	missense
P3	*ZFHX4*	chr8	77766892	77766892	A	C	0.01	p.K2579Q	missense
P4	*ATRX*	chrX	76813026	76813026	A	G	0.37	p.F2199L	missense
P4	*FANCM*	chr14	45605508	45605508	C	T	0.01	p.R92W	missense
P4	*MAD1L1*	chr7	2262335	2262335	G	A	0.04	p.R116W	missense
P5	*TP53*	chr17	7578359	7578369	CCAGCTGCTCA	-	0.67	-	Splice_Site
P5	*ATRX*	chrX	76939973	76939973	C	A	0.71	p.E259X	stopgain
P5	*DCAF4L2*	chr8	88885913	88885913	C	G	0.02	p.G96A	missense
P5	*ZNF536*	chr19	30936480	30936480	C	T	0.03	p.R671W	missense
P6	*CFTR*	chr7	117188841	117188843	GTT	-	0.06	p.452_453 del	nonframeshift deletion
P6	*CSF1R*	chr5	149459730	149459730	C	A	0.15	p.W159C	missense
P6	*CYP2C8*	chr10	96818182	96818182	G	T	0.40	p.Y173X	stopgain
P6	*CYP2C8*	chr10	96818185	96818185	A	C	0.41	p.S172R	missense
P6	*FLCN*	chr17	17120457	17120457	C	T	0.01	p.V368I	missense
P6	*NRG1*	chr8	32621706	32621706	G	T	0.15	p.S413I	missense
P6	*SPG7*	chr16	89614444	89614444	C	T	0.34	p.A529V	missense
P6	*TP53*	chr17	7577022	7577022	G	A	0.58	p.R306X	stopgain
P7	*APC*	chr5	112175631	112175631	A	C	0.06	p.Q1447P	missense
P7	*ATRX*	chrX	76938655	76938655	T	-	0.35	p.K698fs	frameshift deletion
P7	*IGF1R*	chr15	99434653	99434653	C	A	0.01	p.A247D	missense
P7	*KMT2D*	chr12	49445019	49445207	GACAGGTGCGGCTCCTCAGTCTGGGGGGACAGGT GCAATTCCTCAGGCTG	-	0.05	p.753_816 del	nonframeshift deletion
P7	*MDM4*	chr1	204518619	204518619	G	T	0.09	p.E428X	stopgain
P7	*PDGFRB*	chr5	149500488	149500488	T	A	0.07	p.D850V	missense
P7	*RTEL1*	chr20	62323150	62323150	C	T	0.03	p.P871L	missense
P7	*TERT*	chr5	1280332	1280332	G	A	0.04	p.R631W	missense
P8	*RB1*	chr13	48923129	48923129	G	-	0.43	p.V193fs	frameshift deletion
P9	*REG3A*	chr2	79385857	79385857	G	T	0.03	p.R39S	missense
P10	*GABRA2*	chr4	46312273	46312273	C	T	0.07	-	Splice_Site
P10	*PTEN*	chr10	89717609	89717609	G	A	0.23	-	Splice_Site
P10	*PRKAR1A*	chr17	66547263	66547263	T	G	0.08	p.X338E	stoploss
P11	*ASTN2*	chr9	119976939	119976939	C	T	0.01	p.R238H	missense
P11	*CBL*	chr11	119156193	119156193	C	T	0.01	p.L620F	missense
P11	*DUSP27*	chr1	167096241	167096241	C	T	0.01	p.R625W	missense

P12	*ASXL3*	chr18	31323155	31323155	C	A	0.18	p.Q1115K	missense
P12	*CHRM2*	chr7	136700051	136700051	G	C	0.06	p.A147P	missense
P12	*CHRM2*	chr7	136700921	136700921	C	T	0.18	p.P437S	missense
P12	*LRP1B*	chr2	142004917	142004917	T	C	0.23	p.D157G	missense
P12	*MAX*	chr14	65560458	65560458	G	A	0.02	p.R47W	missense
P12	*MDC1*	chr6	30672350	30672472	AGCTCAGGGGCTGCGGGCACAACTGTTTCAGGGG TCTTGACAGAGGACCG	-	0.06	p.1496_1537del	nonframeshift deletion
P12	*NEGR1*	chr1	72163705	72163705	T	G	0.19	p.K218T	missense
P12	*NOTCH3*	chr19	15276290	15276290	G	A	0.02	p.R1902C	missense
P12	*PDHA2*	chr4	96761615	96761615	G	A	0.01	p.G105D	missense
P12	*RYR2*	chr1	237777706	237777706	C	G	0.04	p.R1760G	missense
P12	*TET1*	chr10	70446276	70446276	C	T	0.02	p.P1739L	missense
P12	*TP53*	chr17	7577097	7577097	C	G	0.70	p.D281H	missense
P13	*PRCC*	chr1	156756941	156756941	C	T	0.02	p.A353V	missense
P14	*CSMD3*	chr8	114326955	114326955	T	-	0.19	p.P82fs	frameshift deletion
P14	*ERICH3*	chr1	75037031	75037031	G	A	0.23	p.Q1455X	stopgain
P15	*ASB18*	chr2	237123009	237123009	G	A	0.57	p.P366L	missense
P15	*CFTR*	chr7	117188841	117188843	GTT	-	0.04	p.452_453del	nonframeshift deletion
P15	*INPP4A*	chr2	99163120	99163120	C	T	0.05	p.R376C	missense
P15	*KDM6A*	chrX	44918602	44918602	A	G	0.35	p.Y362C	missense
P15	*SESN1*	chr6	109322606	109322606	G	A	0.25	p.T144M	missense
P16	*CDH9*	chr5	26881525	26881525	A	T	0.15	p.I697N	missense
P16	*ERBB4*	chr2	212568830	212568830	T	G	0.08	p.S430R	missense
P17	*KDR*	chr4	55972909	55972909	C	T	0.08	p.G494E	missense
P17	*LRRTM4*	chr2	77746577	77746577	G	T	0.28	p.L141I	missense
P17	*TP53*	chr17	7578268	7578268	A	C	0.43	p.L194R	missense
P18	*AGO2*	chr8	141568663	141568663	C	G	0.23	p.V267L	missense
P18	*CSMD3*	chr8	113933868	113933868	G	T	0.21	p.P541T	missense
P18	*GEN1*	chr2	17955589	17955589	T	A	0.22	p.L375M	missense
P18	*PIK3CG*	chr7	106508536	106508536	G	T	0.35	p.R177L	missense
P18	*TP53*	chr17	7578222	7578223	TC	-	0.66	p.R209fs	frameshift deletion
P19	*EPHA5*	chr4	66189926	66189926	C	T	0.06	p.R1007Q	missense
P19	*PARP3*	chr3	51979083	51979083	C	T	0.02	p.A235V	missense
P19	*PCDH17*	chr13	58207371	58207371	G	T	0.01	p.V231L	missense
P19	*PER1*	chr17	8053316	8053316	C	T	0.04	p.A168T	missense
P20	*DDR2*	chr1	162724529	162724529	G	A	0.10	p.G101R	missense
P20	*RPL11*	chr1	24019145	24019145	G	A	0.01	p.R18H	missense
P20	*TP53*	chr17	7579518	7579518	C	-	0.22	p.D57fs	frameshift deletion
P21	*ATRX*	chrX	76890150	76890150	T	-	0.38	p.T1582fs	frameshift deletion
P21	*MDC1*	chr6	30673262	30673262	T	C	0.01	p.N1233S	missense
P21	*RYR2*	chr1	237813267	237813267	T	C	0.08	p.F2535L	missense
P21	*SLIT2*	chr4	20591330	20591330	C	T	0.07	p.S1051L	missense

P21	*TP53*	chr17	7578406	7578406	C	T	0.17	p.R175H	missense
P22	*DICER1*	chr14	95569942	95569942	G	A	0.41	p.T1264M	missense
P22	*FLT4*	chr5	180052980	180052980	C	T	0.21	p.R437H	missense
P22	*NCOR1*	chr17	15961376	15961376	C	T	0.11	p.D2005N	missense
P22	*STK19*	chr6	31948509	31948509	C	T	0.04	p.A327V	missense
P22	*TG*	chr8	133912558	133912558	G	A	0.16	p.R1136Q	missense
P23	*TSC2*	chr16	2121510	2121510	G	C	0.02	-	Splice_Site
P24	*SMARCA4*	chr19	11105568	11105568	G	A	0.01	p.G495D	missense
P24	*TYK2*	chr19	10478802	10478802	C	T	0.41	p.A132T	missense
P25	*DAXX*	chr6	33288164	33288164	G	A	0.02	p.S427F	missense
P25	*RYR2*	chr1	237729917	237729917	C	T	0.02	p.R1089C	missense
P25	*TGFBR2*	chr3	30713246	30713246	G	A	0.02	p.V216I	missense
P26	*DAXX*	chr6	33288570	33288570	G	A	0.26	p.R340C	missense
P27	*FANCC*	chr9	97873783	97873783	C	T	0.02	p.G431S	missense
P27	*KMT2B*	chr19	36229048	36229048	C	T	0.01	p.R2610C	missense
P27	*TGFBR2*	chr3	30713579	30713579	G	C	0.11	p.E327Q	missense
P27	*TRRAP*	chr7	98608849	98608849	C	T	0.38	p.L3691F	missense
P27	*TSC2*	chr16	2100463	2100463	C	-	0.59	p.V67fs	frameshift deletion
P28	*RASA1*	chr5	86670656	86670656	G	A	0.21	-	Splice_Site
P28	*EP300*	chr22	41574196	41574196	A	G	0.01	p.M2161V	missense
P28	*FAT3*	chr11	92577119	92577119	C	A	0.03	p.P3529H	missense
P28	*FBN2*	chr5	127607810	127607810	T	G	0.07	p.D2614A	missense
P28	*KMT2D*	chr12	49445439	49445439	T	C	0.04	p.E676G	missense
P28	*LRFN5*	chr14	42356392	42356392	G	T	0.05	p.K188N	missense
P28	*NAV3*	chr12	78334108	78334108	G	C	0.16	p.D85H	missense
P28	*NOTCH3*	chr19	15271586	15271586	C	A	0.03	p.G2285W	missense
P28	*PAK3*	chrX	110437551	110437551	T	G	0.03	p.W339G	missense
P28	*PAK3*	chrX	110437552	110437552	G	T	0.03	p.W339L	missense
P28	*PCDH10*	chr4	134072325	134072325	G	T	0.19	p.V344L	missense
P28	*PKD1*	chr16	2158708	2158708	C	T	0.29	p.V2154I	missense
P28	*PRKAR1A*	chr17	66547263	66547263	T	G	0.06	p.X338E	stoploss
P28	*STT3A*	chr11	125472221	125472221	G	T	0.17	p.R58M	missense
P28	*TEK*	chr9	27203006	27203006	A	C	0.08	p.K657Q	missense
P29	*TSC2*	chr16	2120579	2120579	G	T	0.72	p.Q613H	missense
P30	*IKZF1*	chr7	50467690	50467690	C	A	0.15	p.H180N	missense
P30	*NOTCH2*	chr1	120548047	120548047	T	G	0.11	p.H107P	missense
P30	*PLCG2*	chr16	81971372	81971372	T	A	0.24	p.M1021K	missense
P30	*TSHZ3*	chr19	31770466	31770466	A	C	0.19	p.I78S	missense
P30	*CCND3*								CNV loss
P30	*VEGFA*								CNV loss
P31	*DCAF12L1*	chrX	125686267	125686267	T	C	0.33	p.R109G	missense
P31	*KMT5A*	chr12	123889621	123889621	-	C	0.06	p.C175fs	frameshift insertion
P31	*ZFHX3*	chr16	72832316	72832316	T	C	0.22	p.Y508C	missense

P32	*MST1*	chr3	49722905	49722905	-	GG	0.04	p.P474fs	frameshift insertion
P33	*BCORL1*	chrX	129148426	129148426	G	A	0.37	p.V560M	missense
P34	*MDC1*	chr6	30672875	30672875	T	G	0.01	p.N1362T	missense
P34	*TP53*	chr17	7578190	7578190	T	C	0.59	p.Y220C	missense
P35	*APOB*	chr2	21231020	21231020	C	T	0.03	p.R2907H	missense
P35	*CXCR4*	chr2	136872789	136872789	C	T	0.04	p.A270T	missense
P35	*KCND2*	chr7	119915579	119915579	T	G	0.05	p.F298C	missense
P35	*KMT2A*	chr11	118392074	118392074	C	T	0.02	p.A3862V	missense
P35	*LRP1B*	chr2	141259292	141259292	A	T	0.03	p.C2938X	stopgain
P35	*TP53*	chr17	7576897	7576897	G	A	0.12	p.Q317X	stopgain
P36	*ATRX*	chrX	76939375	76939375	G	C	0.25	p.S458X	stopgain
P36	*CIC*	chr19	42796269	42796269	C	A	0.46	p.T1882K	missense
P36	*CSMD3*	chr8	113697760	113697760	G	A	0.57	p.P786L	missense
P36	*DPYD*	chr1	97915688	97915688	T	A	0.48	p.E611V	missense
P36	*ELF3*	chr1	201980279	201980279	T	G	0.07	p.C5W	missense
P36	*ESR1*	chr6	152265439	152265439	A	G	0.07	p.I298V	missense
P36	*KEL*	chr7	142639969	142639969	G	A	0.46	p.A645V	missense
P36	*LRP1B*	chr2	141113996	141113996	A	C	0.47	p.D3815E	missense
P36	*NLRC5*	chr16	57089379	57089379	G	A	0.43	p.V1232I	missense
P36	*PIK3C2B*	chr1	204394056	204394056	C	T	0.60	p.R1610H	missense
P36	*SMAD4*	chr18	48586278	48586278	A	G	0.54	p.N316S	missense
P36	*SMARCD1*	chr12	50483727	50483727	G	A	0.46	p.V278M	missense
P36	*SOCS1*	chr16	11349292	11349292	G	A	0.42	p.T15I	missense
P36	*UPF1*	chr19	18965420	18965420	T	G	0.06	p.D400E	missense
P36	*ZNF703*	chr8	37553685	37553685	T	A	0.45	p.L63H	missense
P36	*HLA-A*								CNV gain
P36	*MRE11A*								CNV loss
P36	*FANCM*								CNV loss
P37	*CHEK2*	chr22	29121019	29121019	G	A	0.46	p.R180C	missense
P37	*EPHA3*	chr3	89456460	89456460	G	A	0.48	p.A546T	missense
P37	*GRM3*	chr7	86415679	86415679	G	A	0.50	p.V191M	missense
P37	*KEL*	chr7	142658027	142658027	G	A	0.44	p.R130W	missense
P37	*LRP1B*	chr2	141458125	141458125	G	A	0.47	p.R2165W	missense
P37	*MSH2*	chr2	47635594	47635594	T	C	0.52	p.V89A	missense
P37	*NTRK3*	chr15	88799324	88799324	C	A	0.49	p.V21F	missense
P37	*PIK3C2B*	chr1	204394056	204394056	C	T	0.50	p.R1610H	missense
P37	*RASA1*	chr5	86564492	86564492	G	C	0.48	p.G75A	missense
P37	*TMPRSS2*	chr21	42866388	42866388	A	C	0.49	p.Y45D	missense
P37	*STK19*								CNV loss
P38	*ASXL3*	chr18	31324907	31324907	G	T	0.54	p.A1699S	missense
P38	*AXIN2*	chr17	63533904	63533904	G	A	0.58	p.A417V	missense
P38	*BLM*	chr15	91306241	91306241	G	A	0.46	p.R643H	missense
P38	*CD274*	chr9	5463051	5463051	T	G	0.05	p.N90K	missense

P38	*ELF3*	chr1	201980279	201980279	T	G	0.08	p.C5W	missense
P38	*KMT2D*	chr12	49431094	49431094	T	C	0.46	p.M3349V	missense
P38	*KMT2D*	chr12	49445352	49445378	GAGTCCTCAGGTGGTGGGGATGTGGGG	-	0.42	p.696_705 del	nonframeshift deletion
P38	*PHOX2B*	chr4	41748011	41748028	GCCGCCGCCGCTGCCGCG	-	0.02	p.247_253 del	nonframeshift deletion
P38	*SMAD7*	chr18	46476729	46476729	C	A	0.53	p.E22D	missense
P38	*ZNF423*	chr16	49672095	49672095	T	G	0.01	p.H263P	missense
P38	*MLH1*								CNV gain
P39	*ASXL3*	chr18	31322948	31322948	G	A	0.02	p.G1046R	missense
P39	*BAP1*	chr3	52440914	52440914	C	T	0.04	p.G197E	missense
P39	*BBC3*	chr19	47735845	47735845	C	T	0.02	p.M5I	missense
P39	*BLM*	chr15	91295072	91295072	A	T	0.02	p.K285N	missense
P39	*ENG*	chr9	130586720	130586720	T	A	0.38	p.R333W	missense
P39	*EPHA3*	chr3	89448610	89448610	A	T	0.15	p.E525V	missense
P39	*GATA6*	chr18	19762922	19762922	C	T	0.25	p.P513L	missense
P39	*JAK3*	chr19	17945434	17945435	GG	-	0.37	p.V765fs	frameshift deletion
P39	*MAP2K1*	chr15	66729140	66729140	G	T	0.31	p.Q116H	missense
P39	*POLE*	chr12	133226097	133226097	T	C	0.06	p.E1267G	missense
P39	*SDHA*	chr5	233639	233639	G	A	0.05	p.G315R	missense
P39	*SH2B3*	chr12	111856453	111856453	G	C	0.24	p.E168D	missense
P39	*TSHR*	chr14	81609559	81609559	A	G	0.03	p.D386G	missense
P39	*ZNF217*	chr20	52192812	52192812	C	A	0.14	p.G831W	missense
P39	*PAK1*								CNV gain
P39	*NCOR1*								CNV gain
P39	*MDC1*								CNV gain
P39	*HLA-C*								CNV gain
P39	*HLA-B*								CNV gain
P39	*STK19*								CNV gain
P39	*NOTCH4*								CNV gain
P39	*TAP2*								CNV gain
P39	*TAP1*								CNV gain
P39	*DAXX*								CNV gain
P39	*FANCE*								CNV gain
P39	*CDKN1A*								CNV gain
P39	*PIM1*								CNV gain
P39	*CCND3*								CNV gain
P39	*VEGFA*								CNV gain
P39	*MAP2K4*								CNV gain
P40	*FANCB*	chrX	14868673	14868673	C	A	0.02	p.D484Y	missense
P40	*FLT1*	chr13	29001999	29001999	G	A	0.11	p.S389L	missense
P40	*IRF4*	chr6	393215	393215	C	G	0.06	p.N21K	missense
P40	*DNMT1*								CNV gain

P40	*TYK2*								CNV gain
P40	*KEAP1*								CNV gain
P40	*CARM1*								CNV gain
P40	*CALR*								CNV gain
P40	*SMARCA4*								CNV gain
P41	*KMT5A*	chr12	123874011	123874016	GGCGGC	-	0.01	p.14_16del	nonframeshift deletion
P41	*STAT4*	chr2	191927638	191927638	A	T	0.02	p.L264Q	missense
P41	*TET1*	chr10	70405557	70405557	G	A	0.68	p.G1024E	missense
P41	*NCOR1*								CNV gain
P41	*DNAJB1*								CNV gain
P41	*NOTCH3*								CNV gain
P41	*BRD4*								CNV gain
P41	*BCL2L1*								CNV gain
P41	*MYC*								CNV gain
P42	*HLA-A*	chr6	29912042	29912042	G	C	0.22	p.V255L	missense
P43	*CD274*	chr9	5463051	5463051	T	G	0.07	p.N90K	missense
P43	*FAT1*	chr4	187549454	187549454	G	C	0.10	p.T1555R	missense
P43	*NKX3-1*	chr8	23538808	23538808	C	T	0.10	p.V211I	missense
P43	*NOTCH3*	chr19	15271970	15271970	C	T	0.02	p.V2157I	missense
P43	*NOTCH3*	chr19	15271971	15271971	A	C	0.02	p.C2156W	missense
P43	*NCOR1*								CNV gain
P43	*FLCN*								CNV gain
P43	*GID4*								CNV gain
P43	*FANCD2*								CNV loss
P44	*CCND2*	chr12	4409137	4409137	G	A	0.01	p.A278T	missense
P44	*CIC*	chr19	42794844	42794844	G	C	0.30	p.G1551R	missense
P44	*DOT1L*	chr19	2222550	2222550	A	C	0.07	p.N1128H	missense
P44	*GNA11*	chr19	3121157	3121157	A	C	0.07	p.K354Q	missense
P44	*SOX10*	chr22	38369713	38369713	C	T	0.03	p.R397H	missense
P44	*TP53*	chr17	7577539	7577539	G	A	0.88	p.R248W	missense
P45	*NF1*	chr17	29677337	29677337	G	A	0.48	-	Splice_Site
P45	*ATRX*	chrX	76937770	76937770	T	-	0.79	p.K993fs	frameshift deletion
P45	*CASP8*	chr2	202141643	202141643	C	T	0.06	p.P237S	missense
P45	*INPP4A*	chr2	99179978	99179978	G	A	0.42	p.V641M	missense
P45	*KEAP1*	chr19	10600425	10600425	C	-	0.76	p.G477fs	frameshift deletion
P45	*MAP2K1*	chr15	66782067	66782067	A	C	0.06	p.N345T	missense
P45	*MGA*	chr15	41961172	41961172	T	C	0.39	p.I27T	missense
P45	*NSD1*	chr5	176665445	176665445	G	A	0.01	p.V1108M	missense
P45	*PAX5*	chr9	37002705	37002705	T	C	0.08	p.S182G	missense
P45	*RET*	chr10	43622042	43622042	C	T	0.03	p.A1020V	missense
P45	*SETD2*	chr3	47155395	47155395	C	A	0.72	p.W1562C	missense
P45	*SMARCA4*	chr19	11130346	11130346	A	G	0.22	p.Y862C	missense

P45	*TBX3*	chr12	115110067	115110067	G	T	0.01	p.P584H	missense
P46	*TRAF7*	chr16	2225949	2225949	A	C	0.06	p.I581L	missense
P47	*DROSHA*	chr5	31468124	31468124	T	G	0.11	p.N763T	missense
P47	*FAT1*	chr4	187524809	187524809	A	C	0.14	p.V3624G	missense
P47	*FGFR3*	chr4	1808656	1808656	A	C	0.06	p.T757P	missense
P47	*HIST1H3H*	chr6	27778090	27778090	A	C	0.06	p.K80T	missense
P47	*KMT2C*	chr7	151860655	151860655	T	G	0.08	p.Q3336P	missense
P47	*MAP2K2*	chr19	4090607	4090607	T	G	0.07	p.T398P	missense
P47	*PIM1*	chr6	37138106	37138106	A	C	0.11	p.T10P	missense
P47	*RAF1*	chr3	12627205	12627205	T	G	0.08	p.Q504P	missense
P47	*MAPK1*								CNV loss
P47	*MYCL1*								CNV loss
P48	*ALK*	chr2	29449820	29449820	G	A	0.02	p.T1012M	missense
P48	*ALOX12B*	chr17	7979002	7979002	G	A	0.05	p.P522L	missense
P48	*ARAF*	chrX	47424409	47424409	C	T	0.07	p.A110V	missense
P48	*ATR*	chr3	142242842	142242842	G	A	0.06	p.T1382I	missense
P48	*CARM1*	chr19	10982408	10982408	-	GGCGCG GGC	0.08	p.P10delins PGAG	nonframeshift insertion
P48	*CDK12*	chr17	37687498	37687498	T	C	0.02	p.Y1468H	missense
P48	*CDKN2B*	chr9	22008929	22008929	C	T	0.21	p.M8I	missense
P48	*CREBBP*	chr16	3820825	3820825	C	T	0.05	p.G876R	missense
P48	*FAT1*	chr4	187509861	187509861	G	C	0.02	p.A4551G	missense
P48	*FAT1*	chr4	187540683	187540683	A	C	0.05	p.S2353A	missense
P48	*FAT1*	chr4	187557941	187557941	C	T	0.02	p.R1257Q	missense
P48	*FLT4*	chr5	180053001	180053001	G	A	0.02	p.S430F	missense
P48	*GALNT12*	chr9	101570116	101570116	G	A	0.20	p.G46R	missense
P48	*KAT6A*	chr8	41791269	41791269	G	A	0.05	p.P1490L	missense
P48	*KDM5A*	chr12	438072	438072	C	T	0.02	p.A633T	missense
P48	*KEL*	chr7	142641420	142641420	T	A	0.02	p.E494V	missense
P48	*KMT2C*	chr7	151859789	151859789	G	A	0.05	p.P3625S	missense
P48	*KMT2D*	chr12	49434871	49434871	T	C	0.09	p.T2228A	missense
P48	*LATS2*	chr13	21557450	21557450	T	C	0.04	p.I799V	missense
P48	*MGA*	chr15	41962104	41962104	A	G	0.02	p.T338A	missense
P48	*MPL*	chr1	43803863	43803863	C	T	0.07	p.A58V	missense
P48	*MSH2*	chr2	47630353	47630353	C	T	0.02	p.T8M	missense
P48	*MSH3*	chr5	79950724	79950724	-	TCGCAG CGC	0.06	p.A60delin sVAAP	nonframeshift insertion
P48	*MSH6*	chr2	48033981	48033981	-	TTGA	0.04	p.T1225fs	frameshift insertion
P48	*NCOR1*	chr17	15983784	15983784	C	T	0.05	p.R1113Q	missense
P48	*NLRC5*	chr16	57100453	57100453	G	A	0.02	p.E1388K	missense
P48	*PRKD1*	chr14	30068315	30068315	C	T	0.04	p.R703Q	missense
P48	*PRSS1*	chr7	142459834	142459834	C	T	0.02	p.T137M	missense
P48	*REL*	chr2	61118941	61118941	G	A	0.01	p.R45Q	missense

P48	*RHBDF2*	chr17	74475297	74475297	C	T	0.01	p.R141H	missense
P48	*SMARCA4*	chr19	11134252	11134252	G	A	0.02	p.R973Q	missense
P48	*SMARCA4*	chr19	11144146	11144146	C	T	0.01	p.R1243W	missense
P48	*SMARCA4*	chr19	11169019	11169019	G	A	0.01	p.V1505M	missense
P48	*STAT3*	chr17	40469240	40469240	C	T	0.05	p.A702T	missense
P48	*TIPARP*	chr3	156422784	156422784	G	T	0.01	p.S613I	missense
P48	*TRAF7*	chr16	2222282	2222282	G	A	0.21	p.R189Q	missense
P48	*CCND1*								CNV gain
P48	*CDKN2A*								CNV gain
P48	*MUTYH*								CNV loss
P48	*FANCA*								CNV loss
P48	*RHBDF2*								CNV loss
P48	*MYC*								CNV loss
P48	*INPPL1*								CNV loss
P48	*SPG7*								CNV loss
P49	*PMS1*	chr2	190708738	190708738	A	C	0.05	p.M211L	missense
P50	*EML4*	chr2	42528444	42528444	T	G	0.07	p.M518R	missense
P50	*UPF1*	chr19	18964083	18964083	T	G	0.06	p.D371E	missense
P50	*STK19*								CNV gain
P50	*GSTT1*								CNV loss
P51	*GRIN2A*	chr16	9858436	9858436	T	G	0.06	p.N989H	missense
P51	*KMT2D*	chr12	49418693	49418693	A	C	0.07	p.M5274R	missense
P51	*KMT2D*	chr12	49433325	49433325	C	T	0.01	p.A2708T	missense
P51	*LRP1B*	chr2	141250228	141250228	A	C	0.06	p.H3023Q	missense
P51	*NCOR1*	chr17	15995348	15995348	G	A	0.14	p.P949S	missense
P51	*NOTCH4*	chr6	32191659	32191664	AGCAGC	-	0.03	p.14_16del	nonframeshift deletion
P51	*TBX3*	chr12	115109800	115109800	T	C	0.45	p.N673S	missense
P51	*WISP3*	chr6	112389537	112389537	T	G	0.01	p.M240R	missense
P51	*CARM1*								CNV gain
P51	*CALR*								CNV gain
P51	*RB1*								CNV loss
P51	*BRCA2*								CNV loss
P51	*GSTT1*								CNV loss
P52	*ATM*	chr11	108196153	108196153	T	G	0.02	p.I2230S	missense
P52	*DICER1*	chr14	95578574	95578574	A	C	0.05	p.M684R	missense
P52	*TP53*	chr17	7578257	7578257	C	A	0.04	p.E198X	stopgain
P52	*MLH1*								CNV gain
P52	*HIST2H3C*								CNV gain
P52	*GSTT1*								CNV loss
P53	*BRCA1*	chr17	41243538	41243538	T	C	0.03	p.D1337G	missense
P53	*CDKN2C*	chr1	51436103	51436103	-	A	0.23	p.L21fs	frameshift insertion
P53	*FAT3*	chr11	92531050	92531050	C	A	0.18	p.T1624N	missense

P53	*ZFHX3*	chr16	72827813	72827813	G	A	0.10	p.P2009L	missense
P53	*CDKN2A*								CNV loss
P53	*TP53*								CNV loss
P54	*ANK2*	chr4	114275283	114275283	G	A	0.02	p.A1837T	missense
P54	*ZFHX4*	chr8	77766856	77766856	C	A	0.01	p.H2567N	missense
P55	*CTNNB1*	chr3	41266591	41266591	C	T	0.02	p.Q130X	stopgain
P55	*ERBB3*	chr12	56490931	56490931	G	A	0.42	p.G793S	missense
P55	*INPPL1*	chr11	71946941	71946941	G	T	0.02	p.R930S	missense
P55	*PTEN*	chr10	89624258	89624258	-	A	0.08	p.R11fs	frameshift insertion
P55	*TERT*	chr5	1280407	1280407	C	A	0.02	p.V606F	missense
P55	*PIK3C3*								CNV gain
P55	*RIT1*								CNV gain
P55	*NTRK1*								CNV gain
P55	*AKT3*								CNV gain
P55	*NCOA3*								CNV gain
P55	*RINT1*								CNV gain
P55	*PIK3CG*								CNV gain
P55	*CDKN2A*								CNV loss
P55	*CDKN2B*								CNV loss
P55	*CYLD*								CNV loss
P55	*BRCA1*								CNV loss
P55	*PTPRD*								CNV loss
P56	*ATRX*	chrX	76938673	76938673	G	C	0.71	p.S692X	stopgain
P56	*GSK3B*	chr3	119666179	119666179	A	C	0.08	p.M101R	missense
P56	*NBN*	chr8	90982688	90982688	C	T	0.20	p.G267E	missense
P56	*PHOX2B*	chr4	41748011	41748028	GCCGCCGCCGCTGCCGCG	-	0.02	p.247_253del	nonframeshift deletion
P56	*STAT5A*	chr17	40460288	40460288	A	C	0.05	p.I667L	missense
P56	*KDM5A*								CNV gain
P56	*RAD52*								CNV gain
P56	*CCND2*								CNV gain
P56	*FGF23*								CNV gain
P56	*FGF6*								CNV gain
P56	*CHD4*								CNV gain
P56	*RECQL*								CNV gain
P56	*KRAS*								CNV gain
P56	*GLI1*								CNV gain
P56	*CDK4*								CNV gain
P56	*MDM2*								CNV gain
P56	*POLE*								CNV gain
P57	*IRS2*	chr13	110435300	110435302	GGT	-	0.02	p.1033_1034del	nonframeshift deletion
P57	*WEE1*	chr11	9597583	9597583	C	A	0.02	p.S242Y	missense

P58	*RB1*	chr13	48919239	48919239	T	C	0.01	p.L135P	missense
P58	*TP53*	chr17	7579389	7579389	G	A	0.09	p.Q100X	stopgain
P59	*CDK12*	chr17	37627515	37627515	A	T	0.04	p.D477V	missense
P59	*FANCE*	chr6	35430644	35430644	G	T	0.17	p.A488S	missense
P59	*IRS2*	chr13	110435811	110435811	G	T	0.23	p.P864T	missense
P59	*PTPRT*	chr20	41385170	41385170	T	G	0.05	p.K264T	missense
P59	*CCNE1*								CNV gain
P59	*PAX8*								CNV gain
P59	*ERG*								CNV gain
P59	*TMPRSS2*								CNV gain
P59	*ICOSLG*								CNV gain
P59	*SLC19A1*								CNV gain
P59	*STK19*								CNV gain
P59	*NOTCH4*								CNV gain
P59	*RINT1*								CNV gain
P59	*PIK3CG*								CNV gain
P59	*BRAF*								CNV gain
P59	*MYC*								CNV gain
P59	*AGO2*								CNV gain
P60	*ALK*	chr2	30143015	30143015	T	G	0.06	p.S171R	missense
P60	*EPHA7*	chr6	93967209	93967209	C	T	0.03	p.E710K	missense
P60	*ESR1*	chr6	152129336	152129336	T	G	0.09	p.F97V	missense
P60	*FAT3*	chr11	92533650	92533650	T	A	0.01	p.L2491M	missense
P60	*KMT2C*	chr7	151879384	151879384	G	T	0.03	p.P1854H	missense
P60	*MED12*	chrX	70349231	70349231	A	C	0.08	p.I1215L	missense
P60	*NOTCH3*	chr19	15299082	15299082	C	T	0.02	p.D486N	missense
P60	*PIK3C2B*	chr1	204416690	204416690	G	A	0.21	p.S788L	missense
P60	*SUZ12*	chr17	30315391	30315394	TTCG	-	0.85	p.L359fs	frameshift deletion
P60	*RRM2*								CNV gain
P60	*MYCN*								CNV gain
P60	*GEN1*								CNV gain
P60	*FAM135B*								CNV gain
P60	*AGO2*								CNV gain
P60	*RECQL4*								CNV gain
P60	*CHEK1*								CNV gain
P60	*IGF1R*								CNV gain
P60	*PIK3CA*								CNV gain
P60	*SOX2*								CNV gain
P60	*TP63*								CNV gain
P60	*HGF*								CNV gain
P60	*CDK6*								CNV gain
P60	*MET*								CNV gain
P60	*BRAF*								CNV gain

P60	*EZH2*								CNV gain
P60	*MYC*								CNV gain
P60	*TBL1XR1*								CNV gain
P60	*KLHL6*								CNV gain
P60	*MAP3K13*								CNV gain
P60	*ETV5*								CNV gain
P60	*BCL6*								CNV gain
P60	*FGF12*								CNV gain
P60	*MDH2*								CNV gain
P60	*MAGI2*								CNV gain
P61	*AGO2*	chr8	141572702	141572702	C	T	0.02	p.G123D	missense
P61	*PTPRT*	chr20	41385170	41385170	T	G	0.05	p.K264T	missense
P61	*TFE3*								CNV gain
P61	*STAG2*								CNV gain
P61	*SH2D1A*								CNV gain
P61	*BCORL1*								CNV gain
P61	*GPC3*								CNV gain
P61	*RB1*								CNV loss
P61	*AKT3*								CNV loss
P61	*NTRK2*								CNV loss
P62	*ETV6*	chr12	12022817	12022817	A	C	0.07	p.H308P	missense
P62	*EZH1*	chr17	40874915	40874915	A	C	0.05	p.L135V	missense
P62	*LRP1B*	chr2	141072619	141072619	A	C	0.06	p.N4230K	missense
P62	*NOTCH1*	chr9	139396745	139396745	C	T	0.05	p.G1788D	missense
P62	*NTHL1*	chr16	2094696	2094696	C	G	0.11	p.D44H	missense
P62	*RRAS*	chr19	50143330	50143330	G	C	0.06	p.T9R	missense
P62	*SLITRK1*	chr13	84455547	84455547	A	C	0.05	p.N32K	missense
P62	*MAP2K4*								CNV gain
P62	*NCOR1*								CNV gain
P62	*TFE3*								CNV gain
P63	*ASXL3*	chr18	31325601	31325601	A	C	0.02	p.Q1930P	missense
P63	*DPYD*								CNV gain
P63	*LPPR4*								CNV gain
P63	*NCOR1*								CNV gain
P63	*TFE3*								CNV gain
P63	*PTEN*								CNV loss
P63	*RB1*								CNV loss
P63	*CYSLTR2*								CNV loss
P63	*TP53*								CNV loss
P64	*MED12*	chrX	70340820	70340820	G	T	0.15	-	Splice_Site
P64	*TFE3*								CNV gain
P65	*TSC1*	chr9	135779172	135779172	G	A	0.44	p.R692X	stopgain
P65	*MCL1*								CNV gain

P65	*PARP2*								CNV gain
P65	*BCL2L2*								CNV gain
P65	*NCOR1*								CNV gain
P65	*FLCN*								CNV gain
P65	*GID4*								CNV gain
P65	*CCND3*								CNV gain
P65	*VEGFA*								CNV gain
P65	*MYB*								CNV gain
P65	*IFNGR1*								CNV gain
P65	*TNFAIP3*								CNV gain
P65	*TFE3*								CNV gain
P65	*RB1*								CNV loss
P65	*TP53*								CNV loss
P66	*BBC3*	chr19	47725118	47725118	T	G	0.06	p.H209P	missense
P66	*MGA*	chr15	41961113	41961113	T	G	0.01	p.I7M	missense
P66	*MPL*	chr1	43805737	43805737	C	T	0.01	p.L265F	missense
P66	*PCDH17*	chr13	58207704	58207704	A	C	0.06	p.I342L	missense
P66	*PDGFRB*	chr5	149515211	149515211	T	G	0.06	p.T91P	missense
P66	*TFE3*								CNV gain
P67	*LRP1B*	chr2	141777616	141777616	C	A	0.02	p.W615C	missense
P67	*PARP1*	chr1	226574074	226574074	C	A	0.02	p.E263X	stopgain
P67	*CDK4*								CNV gain
P67	*MDM2*								CNV gain
P67	*FRS2*								CNV gain
P67	*CCND3*								CNV gain
P67	*VEGFA*								CNV gain
P67	*TFE3*								CNV gain
P68	*DICER1*	chr14	95572415	95572415	T	G	0.05	p.N984H	missense
P68	*NOTCH3*	chr19	15276256	15276256	G	A	0.40	p.A1913V	missense
P68	*STK11*	chr19	1207048	1207048	A	C	0.06	p.I46L	missense
P68	*BCL2L2*								CNV gain
P68	*ASXL3*								CNV gain
P68	*POLD1*								CNV gain
P68	*PPP2R1A*								CNV gain
P68	*PDGFRA*								CNV gain
P68	*KIT*								CNV gain
P68	*EP300*								CNV gain
P68	*CYP2D6*								CNV gain
P69	*BMPR1A*	chr10	88672019	88672019	A	G	0.02	p.S185G	missense
P69	*CCNE1*	chr19	30314623	30314623	G	C	0.02	p.S376T	missense
P69	*EMSY*	chr11	76234263	76234263	G	T	0.02	p.K584N	missense
P69	*EPHA2*	chr1	16461581	16461581	G	A	0.02	p.T511M	missense
P69	*FLT3*	chr13	28674628	28674628	T	C	0.02	p.D7G	missense

P69	*IRS1*	chr2	227662829	227662829	A	G	0.02	p.M209T	missense
P69	*KDR*	chr4	55974001	55974001	T	C	0.02	p.T439A	missense
P69	*KNSTRN*	chr15	40685797	40685797	A	C	0.03	p.X317S	stoploss
P69	*LIG4*	chr13	108863107	108863107	G	C	0.02	p.S170R	missense
P69	*LRP1B*	chr2	141130580	141130580	G	A	0.02	p.P3589S	missense
P69	*MAPK3*	chr16	30133319	30133319	T	C	0.03	p.Y60C	missense
P69	*MSH2*	chr2	47656972	47656972	C	T	0.02	p.L390F	missense
P69	*MST1R*	chr3	49934014	49934014	C	G	0.02	p.V800L	missense
P69	*NOTCH2*	chr1	120465007	120465007	T	A	0.02	p.I1689F	missense
P69	*NOTCH4*	chr6	32190550	32190550	C	G	0.03	p.Q63H	missense
P69	*PARP3*	chr3	51978529	51978529	C	T	0.02	p.H146Y	missense
P69	*PTPRT*	chr20	41385170	41385170	T	G	0.10	p.K264T	missense
P69	*RECQL4*	chr8	145741851	145741851	G	C	0.03	p.Q218E	missense
P69	*SH2B3*	chr12	111856673	111856673	C	T	0.02	p.P242S	missense
P69	*SMARCD1*	chr12	50483727	50483727	G	A	0.03	p.V278M	missense
P69	*TAP2*	chr6	32796739	32796739	C	T	0.02	p.A669T	missense
P69	*TET2*	chr4	106158215	106158215	C	T	0.02	p.S1039L	missense
P69	*WRN*	chr8	30949362	30949362	G	A	0.02	p.A616T	missense
P70	*AR*	chrX	66766357	66766389	GGCGGCGGCGGCGGCGGCGGCGGCGGCGGCGGC	-	0.03	p.457_467 del	nonframeshift deletion
P70	*FANCE*	chr6	35423961	35423961	A	C	0.07	p.H229P	missense
P70	*NLRC5*	chr16	57060929	57060929	A	C	0.08	p.S692R	missense
P70	*SPEN*	chr1	16260350	16260350	A	C	0.05	p.M2539L	missense
P70	*TEK*	chr9	27203115	27203115	A	C	0.09	p.Q693P	missense
P71	*EP300*	chr22	41568547	41568547	G	C	0.13	p.K1499N	missense
P71	*FAT3*	chr11	92532310	92532310	G	A	0.07	p.R2044H	missense
P71	*SMARCA4*	chr19	11101955	11101955	C	T	0.17	p.Q459X	stopgain
P71	*FLCN*								CNV gain
P71	*GID4*								CNV gain
P71	*INSR*								CNV gain
P71	*CALR*								CNV gain
P72	*KMT2B*	chr19	36214632	36214632	A	G	0.28	-	Splice_Site
P72	*HLA-B*	chr6	31324496	31324496	G	T	0.03	p.N104K	missense
P72	*KIT*	chr4	55593706	55593706	T	C	0.96	p.F591S	missense
P72	*RB1*	chr13	48954189	48954189	G	A	0.23	p.E464K	missense
P72	*TSC1*	chr9	135771657	135771657	T	G	0.06	p.M1154L	missense
P72	*FAM46C*								CNV gain
P72	*HSD3B1*								CNV gain
P72	*NOTCH2*								CNV gain
P72	*PARP2*								CNV gain
P72	*BCL2L2*								CNV gain
P72	*PRKD1*								CNV gain
P72	*NCOR1*								CNV gain

P72	*FLCN*	CNV gain
P72	*GID4*	CNV gain
P72	*FBXW7*	CNV gain
P72	*PRDM1*	CNV gain
P72	*FAM175A*	CNV loss

Table S4. The pathways and genes involved in the 808-cancer-gene panel.

Signaling pathways	Genes involved
Epigenetic	*ASXL1, ATRX, AURKB, BCORL1, CHD2, DNAJB1, DNMT1, DNMT3A, EP300, H3F3A, HIST1H2BD, HIST1H3B, HIST1H3C, HIST1H3D, HIST1H3J, KAT6A, KDM5C, KDM6A, KMT2A, KMT2B, KMT2C, KMT2D, KMT5A, NCOA3, NSD1, PBRM1, PRDM1, RPS24, SETBP1, SETD2, SF3B1, SMARCA4, SMARCB1, SMARCD1, TAF1, TOP2A, TRIM58*
RTK	*ALK, AR, CBL, CSF3R, EML4, EPHA5, EPHA7, ERF, ESR1, FLT1, FLT3, FLT4, INSR, INSRR, IRS1, JAK2, KDR, KIT, MAPK1, MAPK3, NTRK1, NTRK2, NTRK3, PDGFRA, PDGFRB, PGR, PTPRT, RAC1, RET, RHBDF2, ROS1, SHOC2, SOS1, SPRED1, TEK, TLR4, YES1*
PI3K	*AKT1, AKT2, AKT3, DROSHA, EIF4A2, EPHA3, FAM135B, GABRA6, INPP4A, INPP4B, INPPL1, MAPKAP1, MTOR, PDK1, PIK3C2B, PIK3C2G, PIK3C3, PIK3CA, PIK3CB, PIK3CD, PIK3CG, PIK3R1, PIK3R2, PIK3R3, PPP2R1A, PREX2, PRKACA, PRKAR1A, PTEN, RHEB, RICTOR, RPTOR, STK11, TSC1, TSC2, UPF1, WT1*
DNA damage repair	*ATM, ATR, ARID1A, BAP1, BARD1, BLM, BRCA1, BRCA2, BRIP1,*

	CDK12, CHEK1, CHEK2, ERCC1, ERCC2, ERCC3, ERCC4, FANCA, FANCC, FANCD2, FANCE, FANCF, FANCG, FANCL, FANCM, GEN1, MLH1, MSH2, MSH3, MSH6, NBN, NTHL1, PALB2, PARP1, PMS1, PMS2, POLD1, POLE, PRKDC, RAD50, RAD51, RAD51B, RAD51C, RAD51D, WRN, XPA, XPC, XRCC2
Wnt	*AMER1, APC, AXIN1, AXIN2, BCOR, CDC42, CDC73, CFTR, CHD4, CTNNA1, CTNNA2, CTNNB1, FH, GSK3B, KIF2B, LRP1B, LZTR1, PAK6, PCDH10, PCDH17, RNF43, SALL1, SOX17, SOX2, SOX9, TCF7L2*
p53	*ADGRA2, ALOX12B, MDM2, MDM4, PXDNL, RPS6KA4, TP53, TP53BP1*
Cell cycle	*CCND1, CCND2, CCND3, CCNE1, CDK4, CDK6, CDK8, CDKN1A, CDKN1B, CDKN2A, CDKN2B, CDKN2C, ABL2, BCL2L2, BIRC3, BTG1, CASP8, E2F3, ERRFI1, GNA11, GNAS, HNF1A, MED12, NUF2, PTPRD, RB1, STAG2, SYK, TERT, WEE1*
NOTCH	*NOTCH1, NOTCH2, NOTCH3, NOTCH4, KDM5A, SPEN, FBXW7, NCOR1, EGFL7, TMPRSS2*
MAPK	*ARAF, BRAF, BRD4, CREBBP, ETV1, HRAS, HSP90AA1, KRAS, MAP2K1, MAP2K2, MAP2K4, MAP3K1, MAP3K13, MST1R, NF1, NRAS, RAF1, RIT1*

Table S5. Univariate and multivariate analysis of factors associated with DMFS in OS patients.

Characteristics	Parameters	Univariate analysis			Multivariate analysis		
		HR	95% CI	*P* value	HR	95% CI	*P* value
Age	≥ 18 vs < 18	1.09	0.54-2.22	0.808	0.81	0.38-1.74	0.592
Sex	Male vs Female	1.47	0.70-3.10	0.342	1.47	0.64-3.38	0.365
Primary tumor site	Femur vs Other sites	1.10	0.55-2.23	0.786	1.15	0.56-2.37	0.704
DDR mutations/CNVs	Others vs DDR-W/CNV-A	4.41	2.15-9.06	< 0.001	5.41	2.24-13.09	< 0.001

Table S6. Univariate and multivariate analysis of factors associated with EFS in OS patients.

Characteristics	Parameters	Univariate analysis			Multivariate analysis		
		HR	95% CI	*P* value	HR	95% CI	*P* value
Age	≥ 18 vs < 18	1.01	0.50-2.03	0.975	0.80	0.38-1.69	0.562
Sex	Male vs Female	1.53	0.73-3.18	0.294	1.52	0.66-3.45	0.325
Primary tumor site	Femur vs Other site	0.86	0.43-1.72	0.674	1.04	0.51-2.13	0.905
DDR mutations/CNVs	Others vs DDR-W/CNV-A	3.78	1.86-7.65	< 0.001	4.47	1.93-10.37	< 0.001
